# Intrinsic Burst-Blinking Nanographenes for Super-Resolution Bioimaging

**DOI:** 10.1021/jacs.3c11152

**Published:** 2024-01-26

**Authors:** Xingfu Zhu, Qiang Chen, Hao Zhao, Qiqi Yang, Goudappagouda, Márton Gelléri, Sandra Ritz, David Ng, Kaloian Koynov, Sapun H. Parekh, Venkatesh Kumar Chetty, Basant Kumar Thakur, Christoph Cremer, Katharina Landfester, Klaus Müllen, Marco Terenzio, Mischa Bonn, Akimitsu Narita, Xiaomin Liu

**Affiliations:** † Max Planck Institute for Polymer Research, Ackermannweg 10, 55128 Mainz, Germany; ‡ Organic and Carbon Nanomaterials Unit, Okinawa Institute of Science and Technology Graduate University, Kunigami-gun, Okinawa 904-0495, Japan; § Institute of Molecular Biology (IMB), 55128 Mainz, Germany; ∥ Department of Pediatrics III, University Hospital Essen, 45147 Essen, Germany; ⊥ Molecular Neuroscience Unit, Okinawa Institute of Science and Technology Graduate University, Kunigami-gun, Okinawa 904-0495, Japan

## Abstract

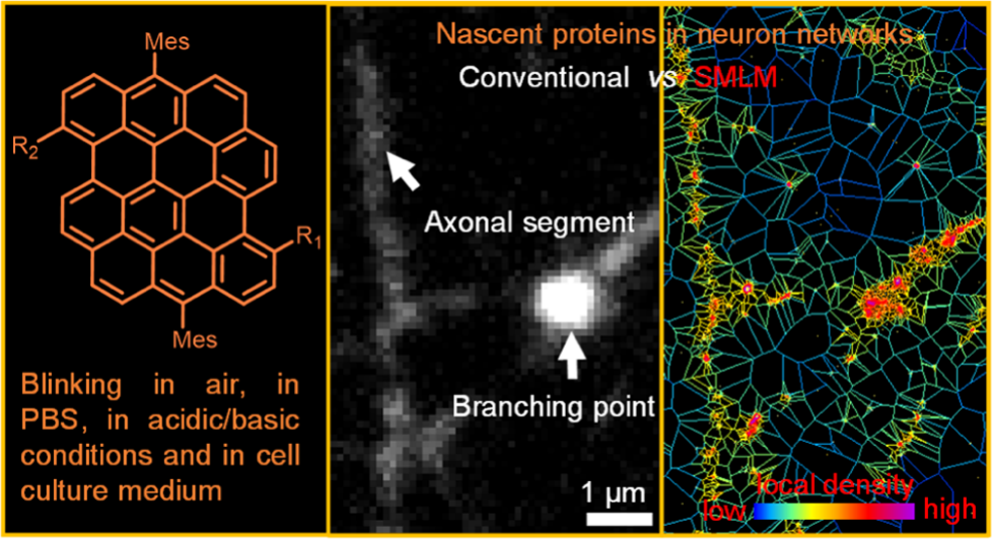

Single-molecule localization microscopy (SMLM) is a powerful technique to achieve super-resolution imaging beyond the diffraction limit. Although various types of blinking fluorophores are currently considered for SMLM, intrinsic blinking fluorophores remain rare at the single-molecule level. Here, we report the synthesis of nanographene-based intrinsic burst-blinking fluorophores for highly versatile SMLM. We image amyloid fibrils in air and in various pH solutions without any additive and lysosome dynamics in live mammalian cells under physiological conditions. In addition, the single-molecule labeling of nascent proteins in primary sensory neurons was achieved with azide-functionalized nanographenes via click chemistry. SMLM imaging reveals higher local translation at axonal branching with unprecedented detail, while the size of translation foci remained similar throughout the entire network. These various results demonstrate the potential of nanographene-based fluorophores to drastically expand the applicability of super-resolution imaging.

## Introduction

Optical super-resolution microscopy (SRM) has emerged as a powerful tool to visualize nanostructures below the optical diffraction limit in life science^1,2^ and material science^3−5^. An increasing number of single-molecule localization microscopy (SMLM) techniques are currently being developed to construct super-resolved images, including photoactivated localization microscopy (PALM),^6^ stochastic optical reconstruction microscopy (STORM),^7^ and second-generation optical super-resolution imaging techniques, i.e., MINFLUX,^8^ SIMFLUX,^9^ and MINSTED.^10^ All of the SRM techniques mentioned above share the same basic principle of separating and localizing adjacent fluorophores in a diffraction-limited area by their different time-dependent behavior, known as blinking. Thus, the development of blinking fluorophores that are able to automatically switch between fluorescent and nonfluorescent states under measurement conditions is key to the improvement of SRM methods.

In addition to organic fluorophores and fluorescent proteins, different types of fluorescent nanoparticles, such as semiconductor quantum dots (QDots), carbon-based nanodots (CDots), polymer dots (PDots), and fluorescent nanodiamonds (FNDs), have been extensively investigated as blinking fluorophores with higher brightness and stability.^11,12^ Among them, CDots have emerged as one of the most promising candidates with unique optical properties that are advantageous for SMLM imaging.^13−17^ CDots can be very small (∼2 and 5 nm) and demonstrate the so-called burst-blinking with a long and complete off state, which are crucial to achieving SRM imaging of high-density labeling samples. Moreover, CDots display buffer-independent fluorescence properties, enabling SRM imaging under a wide range of conditions, such as imaging of materials in air, live-cell imaging under physiological conditions,^13−17^ and potentially correlative light-electron microscopy (CLEM) in vacuum and hydrophobic environments.^18^ Precise control of the chemical structures of CDots, which are typically heterogeneous and mostly undefined at the molecular level, however, remains challenging and constitutes a hurdle for the unambiguous elucidation of their structure–property relationship and fluorescence mechanism,^11,12,17^ thus prohibiting an accurate control of their optical properties. Furthermore, conjugation of CDots to biomolecules remains challenging at the single-molecule level,^11,12,17^ restricting their applicability in bioimaging and biosensing of specific targets.

In this work, we report the synthesis of nanographene-based biocompatible fluorophores for super-resolution bioimaging under a wide range of imaging conditions, which have real-life applications for studying biological systems. Nanographenes, namely, large polycyclic aromatic hydrocarbons with nanoscale graphene structures, can be bottom-up synthesized with atomic precision by synthetic organic chemistry. Some nanographenes have recently been shown to have outstanding burst-blinking properties,^19,20^ similar to CDots, although their application in SRM bioimaging has remained elusive.^21^ Through the decoration of nanographene with hydrophilic side groups, we achieved the SMLM imaging of amyloid fibrils both in air and in various pH solutions. We also imaged lysosome dynamics in live cells under physiological conditions without any additive or irradiation with ultraviolet (UV) light. Finally, we achieved super-resolution imaging of nascent polypeptides in primary sensory neurons using *O*-propargyl-puromycin (OPP) and azide-functionalized nanographenes. Using these data, we were able to document at the single-molecule level how local translation is unevenly distributed along the axonal network, with axonal branching displaying higher levels of translational activity. These results highlight the exciting potential of functionalized nanographenes as intrinsic burst-blinking fluorophores for expanding SRM applications.

## Results and Discussion

### Synthesis and Photophysical Properties of DBOV-OTEG

**Figure 1:**
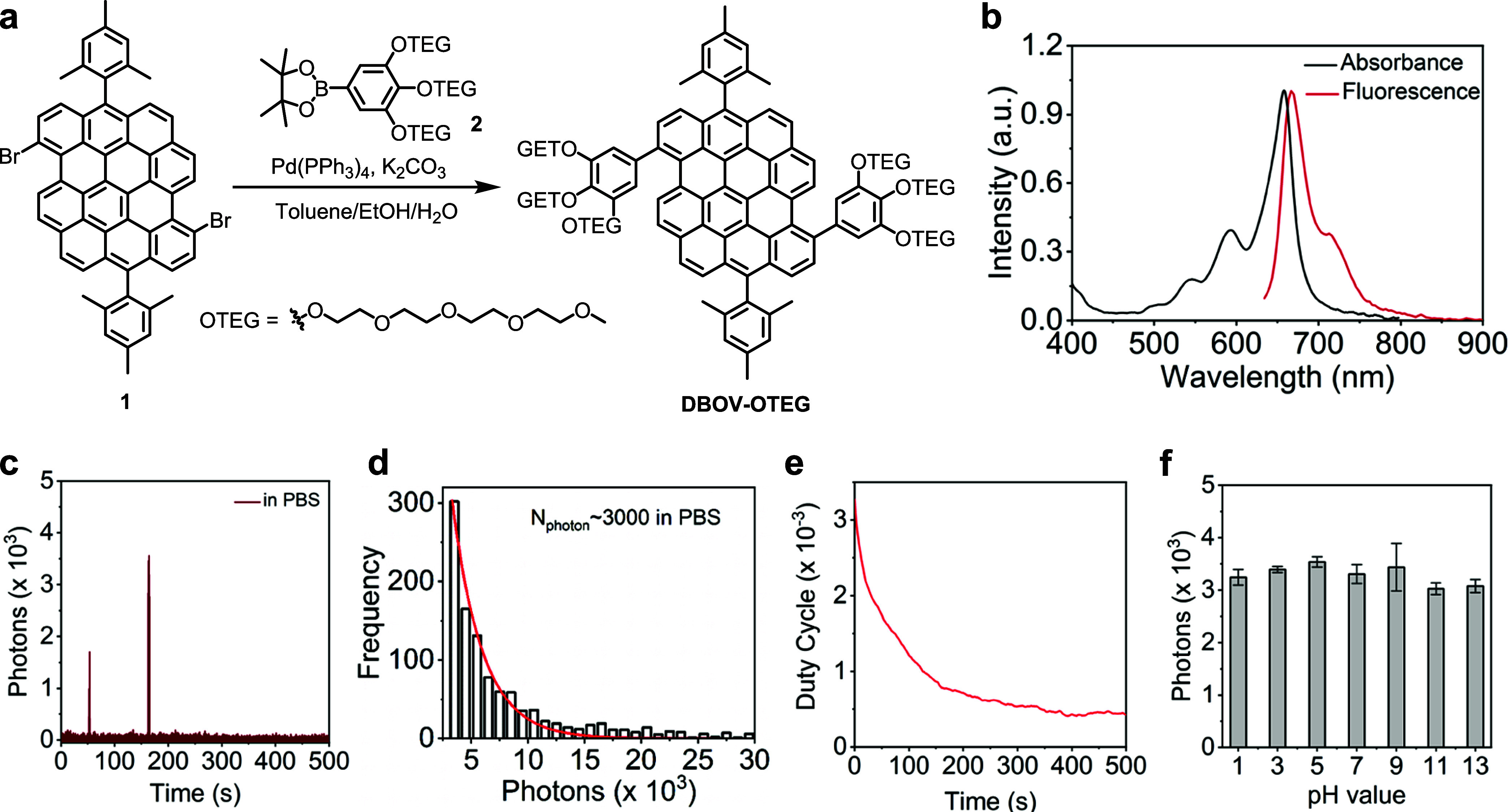
Synthesis and optical characterization of DBOV-OTEG. (a) Chemical structure and synthesis of DBOV-OTEG. (b) UV–vis absorption and emission spectra of DBOV-OTEG in aqueous solution. (c) Single-molecule fluorescence time trace of DBOV-OTEG in PBS solution. (d) Histogram of detected photons per switching event and single-exponential fit of DBOV-OTEG in PBS solution. (e) On–off duty cycle of DBOV-OTEG in PBS solution. (f) Detected photons per switching event of DBOV-OTEG in solutions of various pH.

We chose dibenzo[*hi*,*st*]ovalene (DBOV) as the blinking nanographene for this study, considering its highly stability, well-resolved absorption and emission bands like those of best-performing organic dyes, and red emission with photoluminescence quantum yield of ∼80%.^19,22^ To synthesize hydrophilic and biocompatible nanographenes for the bioimaging, six hydrophilic tetraethylene glycol (TEG) chains were introduced onto the DBOV core (DBOV-OTEG). DBOV-OTEG was synthesized through the Suzuki coupling of dibromo-DBOV **1**(23) and boronic ester **2** in 88% yield (Figure 1a), unambiguously characterized by nuclear magnetic resonance (NMR) spectroscopy and high-resolution mass spectrometry (HRMS) (see the Supporting Information (SI), Figures S1–S9). DBOV-OTEG could be molecularly dissolved in dimethyl sulfoxide (DMSO) as confirmed by fluorescence correlation spectroscopy (FCS) measurements^24^ while the presence of small aggregations with a size of ∼10 nm was indicated in PBS (Figure S18). Since the aggregates are sufficiently small, the impact on the performed SRM imaging is negligible.

UV–visible (UV–vis) absorption and emission spectra of DBOV-OTEG were measured in aqueous solution, exhibiting maxima at 658 and 667 nm with a Stokes shift of 205 cm^–1^ and full width at half-maximum (fwhm) bandwidths of 40 and 38 nm, respectively (Figure 1b). For SMLM imaging, two key blinking properties, photon numbers (detected average photon numbers per blinking event) and on–off duty cycle (fraction of time a molecule resides in its fluorescent state), are crucial for securing high-quality images. While high photon numbers provide better localization precision, a low on–off duty cycle enables better localization accuracy with high labeling density.^25^ Unlike other organic fluorophores that can only blink under optimal blinking buffer conditions or irradiation with UV light, DBOV-OTEG blinks in air and aqueous environments, such as PBS, which are often used in biological applications (Figures 1c and S19). Using single-molecule fluorescence analysis, high photon numbers of ∼3000 per blinking event (Figure 1d) and low on–off duty cycle of 10^–3^ (Figure 1e) with a blinking time of approximately 71 ms were revealed with a 642 nm laser at a laser density of 5 kW/cm^2^, which are comparable to the gold standard Alexa647 under optimized special blinking buffer conditions.^19^

In addition, the blinking properties of DBOV-OTEG were measured over a wide range of pH (from pH 1 to 13) and no obvious change was observed (Figure 1f), indicating that DBOV-OTEG is pH-insensitive and can be used in various pH environments. Unlike most photoswitchable/blinking fluorophores,^26,27^ DBOV-OTEG can, therefore, be used in a wide range of environments, including acidic microenvironment inside lysosomes (pH 4.5–5),^28^ and can withstand sample preparation conditions for hydrogel used in expansion microscopy (pH 7) and surface functionalization of nanocarriers, e.g., for drug delivery (pH 2.7–11).^29,30^

### Nanographenes for SMLM Imaging of Biomaterials in Different Environments

**Figure 2:**
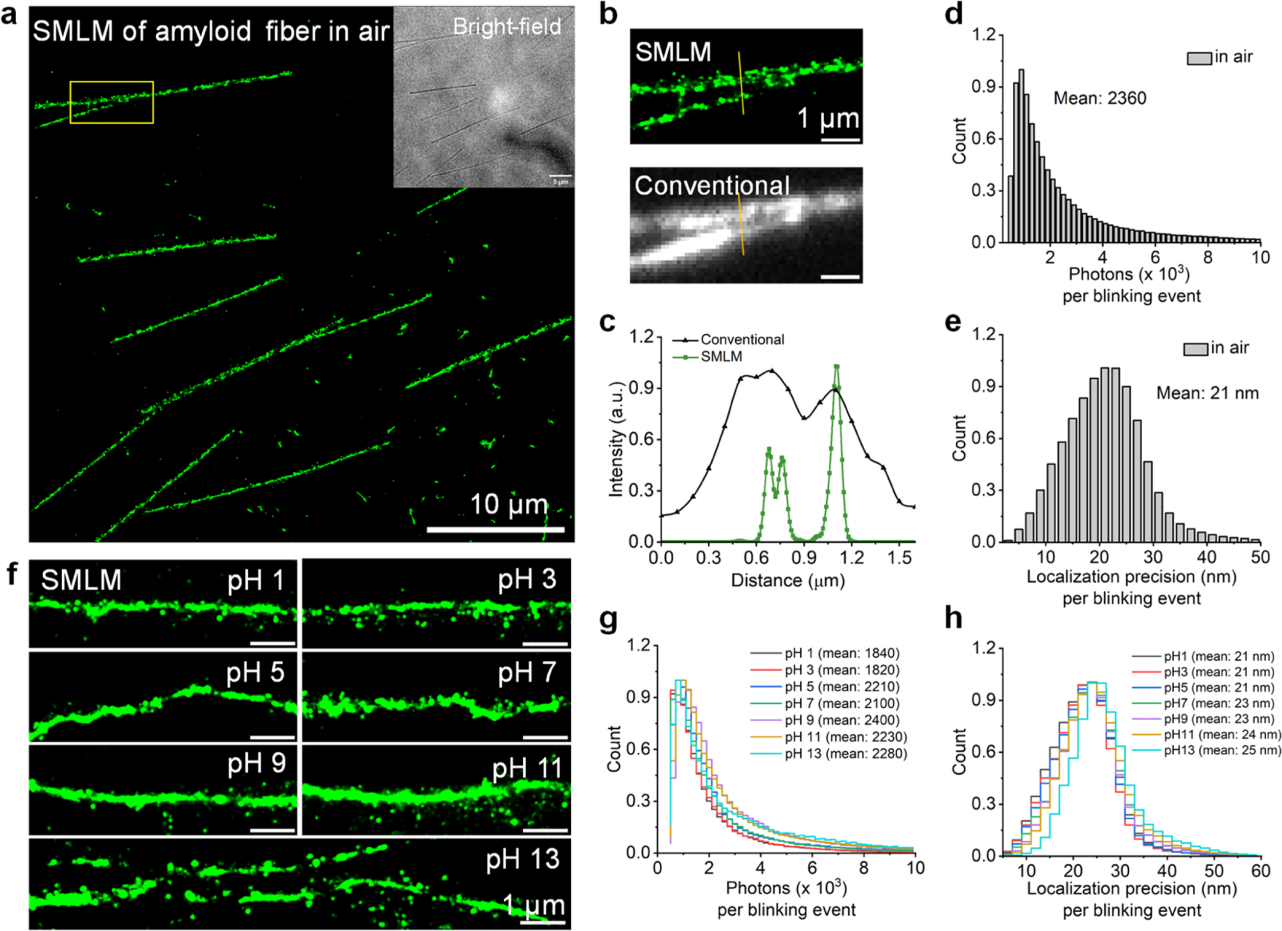
SMLM images of amyloid fibrils labeled with DBOV-OTEG in air and various pH solutions. (a) Reconstructed SMLM image of amyloid fibrils labeled with DBOV-OTEG from 15,000 frames in air. Inset: bright-field image of amyloid fibrils. (b) Magnification of yellow box (top) and the corresponding conventional wide-field fluorescence image (bottom). (c) Cross-line profiles of localization, corresponding regions lined in yellow in (b). (d) Distribution of photon counts per single switching event at 50 ms exposure time in air, with its average value. (e) Distribution of localization precision per single switching event at 50 ms exposure time in air, with its average value. (f) Reconstructed SMLM image of amyloid fibrils from 15,000 frames in various pH solutions. (g) Distribution of photon counts per single switching event at 50 ms exposure time in various pH solutions, with their average values. (h) Distribution of localization precision per single switching event at 50 ms exposure time in various pH solutions, with their average values.

Amyloid fibrils, the aggregates of peptides and proteins, are essential elements in biosystems with various physiological functions.^31^ To demonstrate the robustness of DBOV-OTEG under different environments, we performed SMLM imaging of amyloid fibrils (Aβ1–42) in air as well as in aqueous solutions with various pH values (Figure 2). DBOV-OTEG was conjugated to the amyloid fibrils via physisorption (see the SI for details of sample preparation). The formation of DBOV-OTEG-labeled amyloid fibrils was confirmed by bright-field image (Figure 2a, inset) and verified by co-staining of Thioflavin T (ThT). This commonly used fluorescent dye binds specifically to amyloid fibrils (Figure S20) and showed a good colocalization with DBOV-OTEG (Pearson correlation coefficient = 0.71). SMLM imaging of amyloid fibrils was then performed in air without imaging buffer or illumination with UV light and reconstructed (Figure 2a and Supporting Video 1). SMLM could resolve amyloid fibrils labeled with DBOV-OTEG with high resolution and high signal-to-noise ratio, which are difficult to distinguish in the conventional wide-field image (Figure 2b,c). Note that the SMLM image displays a clear gap (Figure 2b), whereas conventional wide-field image shows a continuous fluorescence signal, which might be due to the on/off time and high density of emitters where the SMLM analysis algorithm sorts out overlapping emitters. The image quality of SMLM is typically limited by the fluorophore’s brightness (number of photons) and on–off duty cycle, together with its labeling density.^25^ The high average photon number of 2360 and a remarkable average localization precision of around 20 nm per frame were achieved at 50 ms exposure time for the SMLM image reconstruction of amyloid fibrils (Figure 2d,e). Furthermore, DBOV-OTEG-labeled amyloid fibrils could be imaged in aqueous solutions of various pH values ranging from pH 1 to 13 (Figure 2f and Supporting Videos 2, 3, 4, 5, 6, 7, and 8) with photon numbers and imaging localization precision comparable to those measured in air (Figure 2g,h). These results demonstrate the versatility of DBOV-OTEG and its advantages over environment-dependent fluorophores, such as Cy5, which requires a special blinking buffer^32^ and spiropyran, which is only applicable in air with UV illumination.^33^

### Nanographenes for SMLM Imaging of Lysosomes in Live Cells

**Figure 3:**
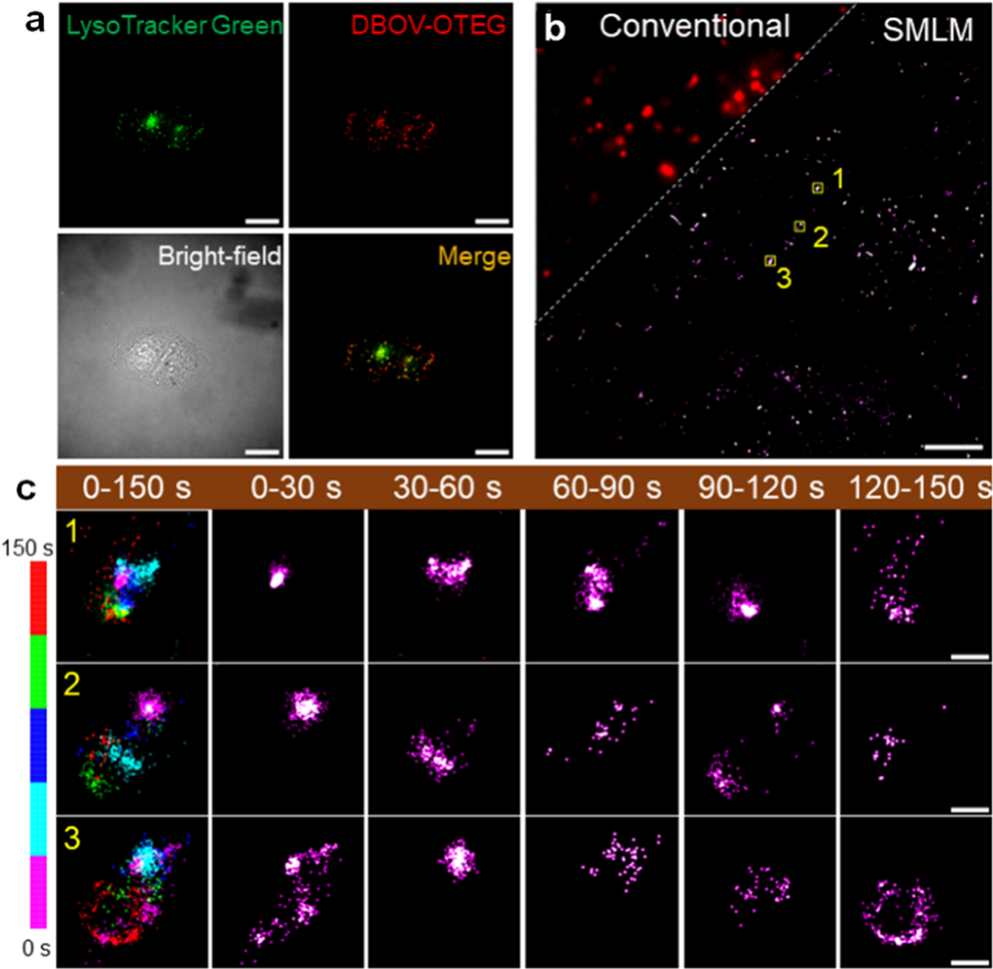
SMLM imaging of lysosomes with DBOV-OTEG in live U2OS cells. (a) Colocalization of DBOV-OTEG and LysoTracker Green. (b) Conventional wide-field fluorescence image of lysosomes and corresponding SMLM image of lysosomes. SMLM imaging was performed in DMEM (supplement 10% FBS) at room temperature, with 642 nm laser of 1 kW/cm^2^ and 23 ms per frame. A total of 6,500 frames were acquired to reconstruct the SMLM image. (c) Time sequence super-resolution images of lysosomes at 30, 60, 90, 120, and 150 s. Three lysosomes were selected in (b), and corresponding SMLM images were reconstructed every 30 s. Scale bars: 20 μm for (a), 5 μm for (b), and 200 nm for (c).

Live-cell super-resolution imaging is critical to studying the dynamic biological processes, avoiding the introduction of structural artifacts due to cell fixation. SMLM is capable of imaging subcellular structures/organelles in living cells with nanoscale resolution as long as their speed of movement is slow compared to the imaging speed. For live-cell SMLM imaging, the fluorophores should have low toxicity and good cell permeability in addition to optimal blinking properties.^21^ We confirmed the low cytotoxicity of DBOV-OTEG using an MTT assay (Figure S21), indicating the possibility of long-term live-cell imaging. DBOV-OTEG was also able to cross the plasma membrane in U2OS cells and selectively accumulated into lysosomes after endocytosis,^21^ which was confirmed by co-labeling with commercial dye LysoTracker Green (Figures 3a and S22). Lysosomes are multifunctional organelles inside cells that play crucial roles in mediating cellular metabolism and signaling,^34^ but their acidic microenvironments (pH 4.5–5) prevent SMLM imaging with the typical pH-sensitive fluorophores. Notably, the pH-independent blinking properties of DBOV-OTEG enabled SMLM imaging of lysosomes in live U2OS cells in a standard cell culture medium without any additives or irradiation with UV light under physiological conditions suitable for live-cell studies (Figure 3b). The dynamic movement as well as the change in morphology of lysosomes were monitored in 30 s time intervals (Figure 3c). The time sequence super-resolution images of three subareas within one cell clearly revealed the diversity of lysosomes’ movements at a nanoscale. These results highlight the advantages of DBOV-OTEG over state-of-the-art lysosome markers for SMLM,^28^ achieving 1.5 times improvement in localization precision and 7 times brighter fluorescence, and a substantially enhanced accuracy in the imaging localization and lysosome dynamics analysis enabled by a lower duty cycle. The pH-independent blinking properties also potentially allow for the simultaneous targeting and imaging of multiple organelles in addition to lysosomes by the proper functionalization of other nanographenes.

### Nanographenes for SMLM Imaging of Global Nascent Proteins in Neurons

**Figure 4:**
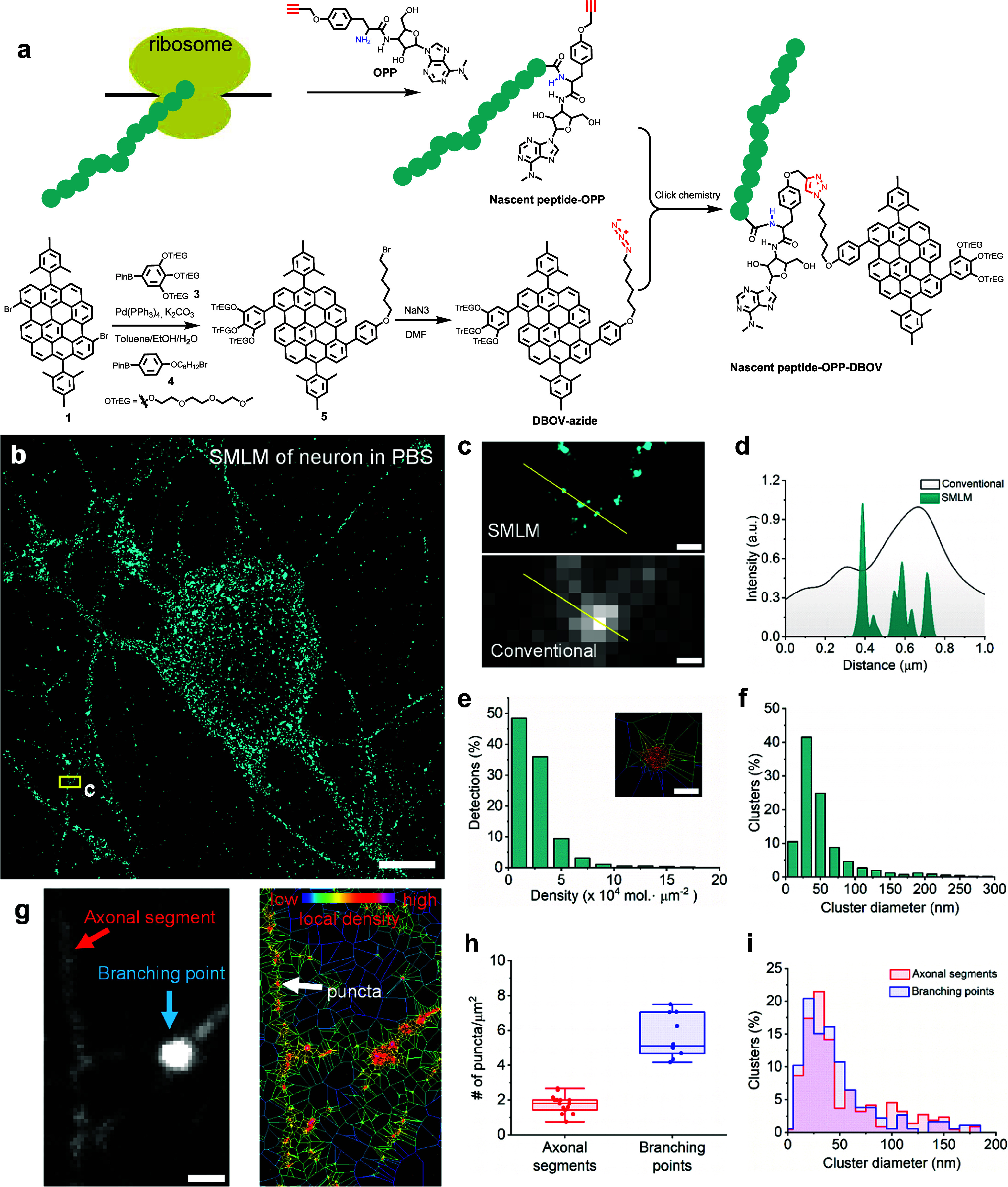
SMLM imaging of global nascent proteins labeled with DBOV-azide in DRG neurons. (a) Reaction schematic illustrating the synthesis of DBOV-azide and the labeling of global nascent proteins in neurons via click chemistry. (b) Reconstructed SMLM image of global nascent proteins in neurons. Imaging was performed in PBS solution. (c) Magnification of SMLM image and conventional wide-field fluorescence image for the yellow box region in (b), respectively. (d) Cross-line profiles of SMLM image and conventional wide-field fluorescence image lined in yellow in (c). (e) Distribution of the first-rank density (single-molecule localizations/μm^2^) of global nascent proteins in (b). The inset shows one representative protein cluster by the Voronoi diagram segment (the red line is the estimated outline of this protein cluster). (f) Cluster size distribution for global nascent proteins in (b) (∼2900 clusters). (g) Conventional wide-field fluorescence image of networks in neurons (left) and corresponding Voronoi diagram image (right) of the same position. The red arrow in (g) (left) indicates the linear axon, and the light blue arrow in (g) (left) shows the branching point (intersection between multiple axons). The white arrow in (g) (right) indicates one puncta in linear axon in reconstructed SMLM image. (h) Number of puncta (cluster)/μm^2^ of axonal segments and branching points in neuron networks. (i) Cluster size distribution of axonal segments and branching points in neuron networks. Scale bar: 5 μm for (b), 1 μm for (g), 200 nm for (c), 50 nm for (e).

Single-molecule imaging of specific targets (e.g., DNA, RNA, proteins) in complex cellular environments is very desirable and allows for unprecedented insights into biological systems. To achieve site-specific labeling of DBOV, we designed DBOV-azide with three triethylene glycol chains to ensure water solubility and an azide residue suitable for the click reaction. For the synthesis of DBOV-azide, dibromo-DBOV **1** was subjected to a Suzuki coupling with two different boronic esters **3** and **4**, which statistically gave DBOV bromide **5** in 25% yield (Figure 4a). DBOV bromide **5** was then reacted with sodium azide to afford DBOV-azide in 85% yield (see the SI for details). The blinking properties of DBOV-azide were found to be similar to those of DBOV-OTEG (Figure S23).

Local protein synthesis is critical in cells with extreme morphology, particularly neurons that transport, localize, and translate mRNAs in axons during axonal development.^35,36^ Local mRNAs are crucial to axonal homeostasis, allowing for fast and localized on-demand translation,^37^ which enables spatial and temporal regulation of the axonal protein content,^38^ and rapid response to external and/or internal stimuli. Thus, axonal protein translation plays a crucial role in axonal development and homeostasis,^39^ as well as in response to stimuli^40^ and nerve injury.^41^ Axonal local translation has also recently risen to prominence in the context of neurodegenerative diseases.^42^ To study axonal translation, biochemical labeling and imaging of nascent synthesized proteins has been performed in neurons and even in vivo in mice,^43,44^ but current fluorescence imaging of nascent proteins based on confocal microscopy is restricted by the diffraction limit.^41^

To gain a deeper insight into the synthesis of nascent proteins by SMLM imaging, we labeled newly synthesized nascent polypeptides in dorsal root ganglia (DRG) sensory neurons via the incorporation of *O*-propargyl-puromycin (OPP)^45^ and subsequent click reaction with DBOV-azide, based on the copper-catalyzed azide–alkyne cycloaddition (CuAAC) (Figure 4a).^46^ Conventional wide-field imaging showed that a fluorescence signal was homogeneously distributed within the neuronal cell body and axons (Figure S24). DBOV-azide on its own did not react with other biomolecules in neurons, confirming the high selectivity of DBOV-azide to the cytosolic protein and OPP. Little labeling could also be observed when neurons were treated with anisomycin, another antibiotic that stops ribosome translation and competes with OPP prior to the treatment with OPP and the subsequent click reaction with DBOV-azide, confirming the high labeling selectivity of OPP to nascent proteins.

One of the unique advantages of SMLM over other fluorescence imaging techniques is its inherent capability to detect individual blinking events of single molecules. These events can be used to investigate, e.g., protein clustering. However, the quantification of exact protein numbers in clusters is difficult due to over- and undercounting of molecules.^47−49^ SMLM imaging of nascent proteins in neurons could be achieved in PBS solution without any additives, providing super-resolved images after the reconstruction (Figure 4b and Supporting Video 9). Notably, SMLM imaging enabled us to clearly distinguish three protein clusters in a branch of an axon, which could not be resolved in its corresponding wide-field image (Figure 4c,d). To obtain a detailed map of nascent proteins in neuronal axons, we further performed the cluster analysis of all localization data based on Voronoi diagrams,^50^ which could effectively segment protein clusters and calculate the local density and diameter of global nascent proteins (Figure 4e,f). The Voronoi cluster analysis revealed a mean cluster size of 50 nm in diameter with an average density of 2.5 × 10^4^ localizations/μm^2^. Although the conventional wide-field fluorescence imaging revealed that the extent of local translation at branch points is greater than the one in axonal fragments between branches (Figure 4g (left) and Figure S25), it could not provide an accurate analysis of the number of translation foci and their cluster size distribution due to the diffraction limit. In contrast, with cluster analysis of SMLM images, the average number of puncta/μm^2^ at the branch points (intersection between multiple axons) could be calculated (5.1 punctas/μm^2^) to be around 2.8 times that of the axonal fragments in between (1.8 punctas/μm^2^, Figure 4h). On the other hand, the cluster size and density of translation foci in branching points are comparable with those puncta in axon fragments (Figures 4i and S25b). These results suggest that local translation at axonal branching is higher in terms of the number of synthesized proteins, while the sizes of translation foci remain similar throughout the network. This technology offers the opportunity to obtain a detailed map of translationally active foci in neuronal axons at a resolution that was not possible before and could help shed new light on the phenomena of axonal translation, which has broad implications on both neuronal injury and neurodegenerative diseases. Taken together, our data demonstrate the potential of DBOV-azide for studying axonal translation and other cellular metabolism.

## Conclusions and Outlook

In summary, hydrophilic, biocompatible, and functionalized nanographenes were synthesized as intrinsic burst-blinking fluorophores for SRM applications, successfully deployed for amyloid fibrils imaging both in air and various pH conditions as well as live-cell imaging under physiological conditions, as a proof of concept. DBOV-OTEG displayed an excellent intrinsic blinking behavior, uncoupled from imaging buffer conditions, irradiation of UV light, and pH, making it suitable for super-resolution imaging in various applications, ranging from materials to live/fixed cell imaging, and with the potential to further explore the relationship of functions and structures of materials as well as the interaction of materials and biosystems. Furthermore, we performed SMLM imaging of global nascent proteins of neurons labeled with DBOV-azide via click chemistry in a PBS solution. The unique cluster analysis of SMLM enabled a detailed map of translationally active foci in neuronal axons at the single-molecule level. This kind of resolution has not been achieved before for the visualization of global local translation in sensory neuron axons, and it allows for much greater mechanistic insights into this biological phenomenon, which is critical to axonal physiology and pathology. Thus, this technology could help us better understand local translation in response to internal and external stimuli.

While DBOV-OTEG and DBOV-azide serve as excellent prototypes of DBOV-based blinking fluorophores, the synthetic protocol that we have established also allows for the introduction of other functional groups for various bioorthogonal reactions from the literature^51^ or conjugation with nanobody or antibody used in immunofluorescence, as well as ligands for specific tags, such as SNAP-tag, Halo-tag, and Clip-tag, for directly targeting specific proteins and subcellular structures in the live-cell SMLM. We also envision that multicolor SMLM imaging using nanographenes is enabled by the readily tunable absorption and emission wavelengths by tuning the spatial extent of the aromatic structures.^19,52^

Besides the conventional SMLM imaging presented here, the intrinsic blinking properties of nanographenes may also be compatible with second-generation optical super-resolution imaging techniques, e.g., MINFLUX, SIMFLUX, and MINSTED, to achieve ultrahigh-precision localization (1–3 nm) of individual molecules. Furthermore, the robust chemical structures and intrinsic blinking properties of nanographenes may contribute to their potential applications in CLEM,^18^ which combines the advantages of optical fluorescence microscopy and electron microscopy in the long term. Overall, the intrinsic burst-blinking fluorophores based on nanographenes have clear advantages and substantially expand new possibilities for super-resolution imaging in materials and life science.

## Supporting Information Available

SMLM imaging of DBOV-OTEG in air (Movie 1) (AVI)
SMLM imaging of DBOV-OTEG in solution of pH 1 (Movie 2) (AVI)
SMLM imaging of DBOV-OTEG in solution of pH 3 (Movie 3) (AVI)
SMLM imaging of DBOV-OTEG in solution of pH 5 (Movie 4) (AVI)
SMLM imaging of DBOV-OTEG in solution of pH 7 (Movie 5) (AVI)
SMLM imaging of DBOV-OTEG in solution of pH 9 (Movie 6) (AVI)
SMLM imaging of DBOV-OTEG in solution of pH 11 (Movie 7) (AVI)
SMLM imaging of DBOV-OTEG in solution of pH 13 (Movie 8) (AVI)
SMLM imaging of DBOV-azide labeled neurons in PBS (Movie 9) (AVI)
Experimental details and data analysis; synthesis and characterizations of new compounds; and NMR spectra (PDF)

## Supplementary Material

ja3c11152_si_001.avi

ja3c11152_si_002.avi

ja3c11152_si_003.avi

ja3c11152_si_004.avi

ja3c11152_si_005.avi

ja3c11152_si_006.avi

ja3c11152_si_007.avi

ja3c11152_si_008.avi

ja3c11152_si_009.avi

## References

[ref1] Sahl S. J.; Hell S. W.; Jakobs S. Fluorescence Nanoscopy in Cell Biology. Nat. Rev. Mol. Cell Biol. 2017, 18 (11), 685–701. 10.1038/nrm.2017.71. 28875992

[ref2] Schermelleh L.; Ferrand A.; Huser T.; Eggeling C.; Sauer M.; Biehlmaier O.; Drummen G. P. C. Super-Resolution Microscopy Demystified. Nat. Cell Biol. 2019, 21 (1), 72–84. 10.1038/s41556-018-0251-8. 30602772

[ref3] Pujals S.; Feiner-Gracia N.; Delcanale P.; Voets I.; Albertazzi L. Super-Resolution Microscopy as a Powerful Tool to Study Complex Synthetic Materials. Nat. Rev. Chem. 2019, 3 (2), 68–84. 10.1038/s41570-018-0070-2.

[ref4] Wöll D.; Flors C. Super-Resolution Fluorescence Imaging for Materials Science. Small Methods 2017, 1 (10), 1700191 10.1002/smtd.201700191.

[ref5] Chen T.; Dong B.; Chen K.; Zhao F.; Cheng X.; Ma C.; Lee S.; Zhang P.; Kang S. H.; Ha J. W.; Xu W.; Fang N. Optical Super-Resolution Imaging of Surface Reactions. Chem. Rev. 2017, 117 (11), 7510–7537. 10.1021/acs.chemrev.6b00673. 28306243

[ref6] Betzig E.; Patterson G. H.; Sougrat R.; Lindwasser O. W.; Olenych S.; Bonifacino J. S.; Davidson M. W.; Lippincott-Schwartz J.; Hess H. F. Imaging Intracellular Fluorescent Proteins at Nanometer Resolution. Science 2006, 313 (5793), 1642–1645. 10.1126/science.1127344. 16902090

[ref7] Rust M. J.; Bates M.; Zhuang X. Sub-Diffraction-Limit Imaging by Stochastic Optical Reconstruction Microscopy (STORM). Nat. Methods 2006, 3 (10), 793–795. 10.1038/nmeth929. 16896339 PMC2700296

[ref8] Balzarotti F.; Eilers Y.; Gwosch K. C.; Gynnå A. H.; Westphal V.; Stefani F. D.; Elf J.; Hell S. W. Nanometer Resolution Imaging and Tracking of Fluorescent Molecules with Minimal Photon Fluxes. Science 2017, 355 (6325), 606–612. 10.1126/science.aak9913. 28008086

[ref9] Cnossen J.; Hinsdale T.; Thorsen R. Ø.; Siemons M.; Schueder F.; Jungmann R.; Smith C. S.; Rieger B.; Stallinga S. Localization Microscopy at Doubled Precision with Patterned Illumination. Nat. Methods 2020, 17 (1), 59–63. 10.1038/s41592-019-0657-7. 31819263 PMC6989044

[ref10] Weber M.; Leutenegger M.; Stoldt S.; Jakobs S.; Mihaila T. S.; Butkevich A. N.; Hell S. W. MINSTED Fluorescence Localization and Nanoscopy. Nat. Photonics 2021, 15 (5), 361–366. 10.1038/s41566-021-00774-2. 33953795 PMC7610723

[ref11] Li W.; Schierle G. S. K.; Lei B.; Liu Y.; Kaminski C. F. Fluorescent Nanoparticles for Super-Resolution Imaging. Chem. Rev. 2022, 122 (15), 12495–12543. 10.1021/acs.chemrev.2c00050. 35759536 PMC9373000

[ref12] Jin D.; Xi P.; Wang B.; Zhang L.; Enderlein J.; Van Oijen A. M. Nanoparticles for Super-Resolution Microscopy and Single-Molecule Tracking. Nat. Methods 2018, 15 (6), 415–423. 10.1038/s41592-018-0012-4. 29808018

[ref13] He H.; Liu X.; Li S.; Wang X.; Wang Q.; Li J.; Wang J.; Ren H.; Ge B.; Wang S.; Zhang X.-D.; Huang F. High-Density Super-Resolution Localization Imaging with Blinking Carbon Dots. Anal. Chem. 2017, 89 (21), 11831–11838. 10.1021/acs.analchem.7b03567. 28976184

[ref14] He H.; Chen X.; Feng Z.; Liu L.; Wang Q.; Bi S. Nanoscopic Imaging of Nucleolar Stress Enabled by Protein-Mimicking Carbon Dots. Nano Lett. 2021, 21 (13), 5689–5696. 10.1021/acs.nanolett.1c01420. 34181434

[ref15] Mao J.; Xue M.; Guan X.; Wang Q.; Wang Z.; Qin G.; He H. Near-Infrared Blinking Carbon Dots Designed for Quantitative Nanoscopy. Nano Lett. 2023, 23 (1), 124–131. 10.1021/acs.nanolett.2c03711. 36579734

[ref16] Ye Z.; Wei L.; Geng X.; Wang X.; Li Z.; Xiao L. Mitochondrion-Specific Blinking Fluorescent Bioprobe for Nanoscopic Monitoring of Mitophagy. ACS Nano 2019, 13 (10), 11593–11602. 10.1021/acsnano.9b05354. 31592641

[ref17] Sun X.; Mosleh N. Fluorescent Carbon Dots for Super-Resolution Microscopy. Materials 2023, 16 (3), 890 10.3390/ma16030890. 36769896 PMC9917526

[ref18] Sochacki K. A.; Shtengel G.; Van Engelenburg S. B.; Hess H. F.; Taraska J. W. Correlative Super-Resolution Fluorescence and Metal-Replica Transmission Electron Microscopy. Nat. Methods 2014, 11 (3), 305–308. 10.1038/nmeth.2816. 24464288 PMC3943662

[ref19] Liu X.; Chen S. Y.; Chen Q.; Yao X.; Gelléri M.; Ritz S.; Kumar S.; Cremer C.; Landfester K.; Müllen K.; Parekh S. H.; Narita A.; Bonn M. Nanographenes: Ultrastable, Switchable, and Bright Probes for Super-Resolution Microscopy. Angew. Chem., Int. Ed. 2020, 59 (1), 496–502. 10.1002/anie.201909220. PMC6972658 31657497

[ref20] Jin E.; Yang Q.; Ju C. W.; Chen Q.; Landfester K.; Bonn M.; Müllen K.; Liu X.; Narita A. A Highly Luminescent Nitrogen-Doped Nanographene as an Acid- And Metal-Sensitive Fluorophore for Optical Imaging. J. Am. Chem. Soc. 2021, 143 (27), 10403–10412. 10.1021/jacs.1c04880. 34224242 PMC8283754

[ref21] Lin H. A.; Sato Y.; Segawa Y.; Nishihara T.; Sugimoto N.; Scott L. T.; Higashiyama T.; Itami K. A Water-Soluble Warped Nanographene: Synthesis and Applications for Photoinduced Cell Death. Angew. Chem., Int. Ed. 2018, 57 (11), 2874–2878. 10.1002/anie.201713387. 29380493

[ref22] Chen Q.; Thoms S.; Stöttinger S.; Schollmeyer D.; Müllen K.; Narita A.; Basché T. Dibenzo[ Hi, St]Ovalene as Highly Luminescent Nanographene: Efficient Synthesis via Photochemical Cyclodehydroiodination, Optoelectronic Properties, and Single-Molecule Spectroscopy. J. Am. Chem. Soc. 2019, 141 (41), 16439–16449. 10.1021/jacs.9b08320. 31589425

[ref23] Chen Q.; Wang D.; Baumgarten M.; Schollmeyer D.; Müllen K.; Narita A. Regioselective Bromination and Functionalization of Dibenzo[Hi,St]Ovalene as Highly Luminescent Nanographene with Zigzag Edges. Chem. - Asian J. 2019, 14 (10), 1703–1707. 10.1002/asia.201801822. 30775845

[ref24] Schmitt S.; Nuhn L.; Barz M.; Butt H. J.; Koynov K. Shining Light on Polymeric Drug Nanocarriers with Fluorescence Correlation Spectroscopy. Macromol. Rapid Commun. 2022, 43 (12), 2100892 10.1002/marc.202100892. 35174569

[ref25] Dempsey G. T.; Vaughan J. C.; Chen K. H.; Bates M.; Zhuang X. Evaluation of Fluorophores for Optimal Performance in Localization-Based Super-Resolution Imaging. Nat. Methods 2011, 8 (12), 1027–1040. 10.1038/nmeth.1768. 22056676 PMC3272503

[ref26] Szczurek A.; Klewes L.; Xing J.; Gourram A.; Birk U.; Knecht H.; Dobrucki J. W.; Mai S.; Cremer C. Imaging Chromatin Nanostructure with Binding-Activated Localization Microscopy Based on DNA Structure Fluctuations. Nucleic Acids Res. 2017, 45 (8), e56 10.1093/nar/gkw1301. 28082388 PMC5416826

[ref27] Uno S. N.; Kamiya M.; Yoshihara T.; Sugawara K.; Okabe K.; Tarhan M. C.; Fujita H.; Funatsu T.; Okada Y.; Tobita S.; Urano Y. A Spontaneously Blinking Fluorophore Based on Intramolecular Spirocyclization for Live-Cell Super-Resolution Imaging. Nat. Chem. 2014, 6 (8), 681–689. 10.1038/nchem.2002. 25054937

[ref28] Qiao Q.; Liu W.; Chen J.; Wu X.; Deng F.; Fang X.; Xu N.; Zhou W.; Wu S.; Yin W.; Liu X.; Xu Z. An Acid-Regulated Self-Blinking Fluorescent Probe for Resolving Whole-Cell Lysosomes with Long-Term Nanoscopy. Angew. Chem., Int. Ed. 2022, 61 (21), e202202961 10.1002/anie.202202961. 35263485

[ref29] Zwettler F. U.; Reinhard S.; Gambarotto D.; Bell T. D. M.; Hamel V.; Guichard P.; Sauer M. Molecular Resolution Imaging by Post-Labeling Expansion Single-Molecule Localization Microscopy (Ex-SMLM). Nat. Commun. 2020, 11 (1), 3388 10.1038/s41467-020-17086-8. 32636396 PMC7340794

[ref30] Tonigold M.; Simon J.; Estupiñán D.; Kokkinopoulou M.; Reinholz J.; Kintzel U.; Kaltbeitzel A.; Renz P.; Domogalla M. P.; Steinbrink K.; Lieberwirth I.; Crespy D.; Landfester K.; Mailänder V. Pre-Adsorption of Antibodies Enables Targeting of Nanocarriers despite a Biomolecular Corona. Nat. Nanotechnol. 2018, 13 (9), 862–869. 10.1038/s41565-018-0171-6. 29915272

[ref31] Knowles T. P. J.; Vendruscolo M.; Dobson C. M. The Amyloid State and Its Association with Protein Misfolding Diseases. Nat. Rev. Mol. Cell Biol. 2014, 15 (6), 384–396. 10.1038/nrm3810. 24854788

[ref32] Albertazzi L.; Van Der Zwaag D.; Leenders C. M. A.; Fitzner R.; Van Der Hofstad R. W.; Meijer E. W. Probing Exchange Pathways in One-Dimensional Aggregates with Super-Resolution Microscopy. Science 2014, 344 (6183), 491–495. 10.1126/science.1250945. 24786073

[ref33] Yan J.; Zhao L.; Li C.; Hu Z.; Zhang G.; Chen Z.-Q.; Chen T.; Huang Z.; Zhu J.; Zhu M. Optical Nanoimaging for Block Copolymer Self-Assembly. J. Am. Chem. Soc. 2015, 137 (7), 2436–2439. 10.1021/ja512189a. 25668069

[ref34] Lawrence R. E.; Zoncu R. The Lysosome as a Cellular Centre for Signalling, Metabolism and Quality Control. Nat. Cell Biol. 2019, 21 (2), 133–142. 10.1038/s41556-018-0244-7. 30602725

[ref35] Glock C.; Heumüller M.; Schuman E. M. MRNA Transport & Local Translation in Neurons. Curr. Opin. Neurobiol. 2017, 45, 169–177. 10.1016/j.conb.2017.05.005. 28633045

[ref36] Rangaraju V.; tom Dieck S.; Schuman E. M. Local Translation in Neuronal Compartments: How Local Is Local?. EMBO Rep. 2017, 18 (5), 693–711. 10.15252/embr.201744045. 28404606 PMC5412868

[ref37] Vargas J. N. S.; Sleigh J. N.; Schiavo G. Coupling Axonal MRNA Transport and Local Translation to Organelle Maintenance and Function. Curr. Opin. Cell Biol. 2022, 74, 97–103. 10.1016/j.ceb.2022.01.008. 35220080 PMC10477965

[ref38] Jung H.; Gkogkas C. G.; Sonenberg N.; Holt C. E. Remote Control of Gene Function by Local Translation. Cell 2014, 157 (1), 26–40. 10.1016/j.cell.2014.03.005. 24679524 PMC3988848

[ref39] Batista A. F. R.; Martínez J. C.; Hengst U. Intra-Axonal Synthesis of SNAP25 Is Required for the Formation of Presynaptic Terminals. Cell Rep. 2017, 20 (13), 3085–3098. 10.1016/j.celrep.2017.08.097. 28954226 PMC5659736

[ref40] Graber T. E.; Hébert-Seropian S.; Khoutorsky A.; David A.; Yewdell J. W.; Lacaille J. C.; Sossin W. S. Reactivation of Stalled Polyribosomes in Synaptic Plasticity. Proc. Natl. Acad. Sci. U.S.A. 2013, 110 (40), 16205–16210. 10.1073/pnas.1307747110. 24043809 PMC3791775

[ref41] Terenzio M.; Koley S.; Samra N.; Rishal I.; Zhao Q.; Sahoo P. K.; Urisman A.; Marvaldi L.; Oses-prieto J. A.; Forester C.; Gomes C.; Kalinski A. L.; Pizio A.; Di; Doron-mandel E.; Perry R. B.; Koppel I.; Twiss J. L.; Burlingame A. L.; Fainzilber M. Locally Translated MTOR Controls Axonal Local Translation in Nerve Injury. Science 2018, 359 (6382), 1416–1421. 10.1126/science.aan1053. 29567716 PMC6501578

[ref42] Nagano S.; Araki T. Axonal Transport and Local Translation of MRNA in Neurodegenerative Diseases. Front. Mol. Neurosci. 2021, 14, 697973 10.3389/fnmol.2021.697973. 34194300 PMC8236635

[ref43] Holt C. E.; Martin K. C.; Schuman E. M. Local Translation in Neurons: Visualization and Function. Nat. Struct. Mol. Biol. 2019, 26 (7), 557–566. 10.1038/s41594-019-0263-5. 31270476

[ref44] Koppel I.; Fainzilber M. Omics Approaches for Subcellular Translation Studies. Mol. Omics 2018, 14 (6), 380–388. 10.1039/C8MO00172C. 30338329

[ref45] Liu J.; Xu Y.; Stoleru D.; Salic A. Imaging Protein Synthesis in Cells and Tissues with an Alkyne Analog of Puromycin. Proc. Natl. Acad. Sci. U.S.A. 2012, 109 (2), 413–418. 10.1073/pnas.1111561108. 22160674 PMC3258597

[ref46] Hein J. E.; Fokin V. V. Copper-Catalyzed Azide-Alkyne Cycloaddition (CuAAC) and beyond: New Reactivity of Copper(i) Acetylides. Chem. Soc. Rev. 2010, 39 (4), 1302–1315. 10.1039/b904091a. 20309487 PMC3073167

[ref47] Baumgart F.; Arnold A. M.; Leskovar K.; Staszek K.; Fölser M.; Weghuber J.; Stockinger H.; Schütz G. J. Varying Label Density Allows Artifact-Free Analysis of Membrane-Protein Nanoclusters. Nat. Methods 2016, 13 (8), 661–664. 10.1038/nmeth.3897. 27295310 PMC6404959

[ref48] Annibale P.; Scarselli M.; Kodiyan A.; Radenovic A. Photoactivatable Fluorescent Protein MEos2 Displays Repeated Photoactivation after a Long-Lived Dark State in the Red Photoconverted Form. J. Phys. Chem. Lett. 2010, 1 (9), 1506–1510. 10.1021/jz1003523.

[ref49] Sengupta P.; Jovanovic-Talisman T.; Skoko D.; Renz M.; Veatch S. L.; Lippincott-Schwartz J. Probing Protein Heterogeneity in the Plasma Membrane Using PALM and Pair Correlation Analysis. Nat. Methods 2011, 8 (11), 969–975. 10.1038/nmeth.1704. 21926998 PMC3400087

[ref50] Levet F.; Hosy E.; Kechkar A.; Butler C.; Beghin A.; Choquet D.; Sibarita J. SR-Tesseler: A Method to Segment and Quantify Localization-Based Super-Resolution Microscopy Data. Nat. Methods 2015, 12 (11), 1065–1071. 10.1038/nmeth.3579. 26344046

[ref51] Jewett J. C.; Bertozzi C. R. Cu-Free Click Cycloaddition Reactions in Chemical Biology. Chem. Soc. Rev. 2010, 39 (4), 1272–1279. 10.1039/b901970g. 20349533 PMC2865253

[ref52] Gu Y.; Wu X.; Gopalakrishna T. Y.; Phan H.; Wu J. Graphene-like Molecules with Four Zigzag Edges. Angew. Chem., Int. Ed. 2018, 57 (22), 6541–6545. 10.1002/anie.201802818. 29655220

