# Supplementary material for: Intrinsic Burst-Blinking Nanographenes for Super-Resolution Bioimaging
Source: J Am Chem Soc. 2024 Jan 26;146(8):5195–203. doi: 10.1021/jacs.3c11152 (PMC10910517; doi:10.1021/jacs.3c11152)
Supplement: Supplementary file 10 — ja3c11152_si_010.pdf [file ja3c11152_si_010.pdf]

Supporting information

for

Intrinsic Burst-blinking Nanographenes for Super-resolution Biomaging

Xingfu Zhu,[†,#] Qiang Chen,[†,#] Hao Zhao,[+,#] Qiqi Yang,[†] Goudappagouda,[+] Márton Gelléri,[¶] Sandra Ritz,[¶] David Ng,[†] Kaloian Koynov,[†] Sapun H. Parekh,[†] Venkatesh Kumar Chetty,[§] Basant Kumar Thakur,[§] Christoph Cremer,[†,¶] Katharina Landfester,[†] Klaus Müllen,[†] Marco Terenzio,[‡] Mischa Bonn,[†,*] Akimitsu Narita, [†,+,*] Xiaomin Liu[†,*]

[†]Max Planck Institute for Polymer Research, Ackermannweg 10, 55128 Mainz, Germany;
[+]Organic and Carbon Nanomaterials Unit, Okinawa Institute of Science and Technology Graduate University, Kunigami-gun, Okinawa 904-0495, Japan;
[¶]Institute of Molecular Biology (IMB), 55128 Mainz, Germany;
[§]Department of Pediatrics III, University Hospital Essen, 45147 Essen, Germany;
[‡]Molecular Neuroscience Unit, Okinawa Institute of Science and Technology Graduate University, Kunigami-gun, Okinawa 904-0495, Japan.
[#]These authors contributed equally: Xingfu Zhu, Qiang Chen, Hao Zhao

Correspondence and requests for materials should be addressed to M.B. (email: bonn@mpip mainz.mpg.de), A.N. (email: akimitsu.narita@oist.jp). and X.L. (liuxiaomin@mpip mainz.mpg.de)

Table of Content

Supplementary methods Page 3

Synthesis of DBOV-OTEG and DBOV-azide Page 4

Sample preparations for single-molecule measurements Page 8

Preparation of Aβ fiber Page 9

Live cell SMLM imaging of lysosomes Page 9

Preparation of neurons sample and imaging Page 10

SMLM imaging and data analysis Page 10

^{1}H and ^{13}C NMR for all compounds Page 12

Additional supplementary figures Page 21

References Page 27

Supplementary Methods

Animal experiments

All experiments involving animal subjects were carried out in accordance with the guidelines and regulations of Okinawa Institute of Science and Technology Graduate University (OIST) and approved by OIST Animal Care and Use Committee (Protocol No. 2022-373). Adult (8-10 weeks) male wild type C57BL/6 mice were purchased from Charles River, Japan. Animals were housed at 24.0 ± 0.5°C with alternating 12 hr. day/night cycles and allowed access to food and water ad libitum.

Material and general information

All reactions working with air- or moisture-sensitive compounds were carried out under an argon atmosphere using standard Schlenk line techniques. Unless otherwise noted, all starting materials were purchased from commercial sources and used without further purification. All other reagents were used as received. Thin layer chromatography (TLC) was done on silica gel-coated aluminum sheets with F254 indicator and column chromatography separation was performed with silica gel (particle size 0.063 – 0.200 mm). Nuclear magnetic resonance (NMR) spectra were recorded using Bruker 300, Bruker 400, Bruker 500 and Bruker 700 MHz NMR spectrometers. Chemical shifts (δ) were expressed in ppm relative to the residual of solvents (dichloromethane-d_2, ^{1}H: 5.32 ppm, ^{13}C: 54.00 ppm; chloroform-d, ^{1}H: 7.26 ppm, ^{13}C: 77.16 ppm). Abbreviations: s = singlet, d = doublet, t = triplet, q = quartet, m = multiplet. Coupling constants (J) were recorded in Hertz. High-resolution mass (HR-MS) determinations were carried out on a G6545A Q-ToF (Agilent GmbH, Waldbronn, Germany) with electrospray ionization (ESI). Sample inlet was a 1260 Infinity II HPLC system (Agilent GmbH, Waldbronn, Germany) with G7111B 1260 Quaternary Pump, G7129A 1260 Vialsampler and G7116A 1260 Multicolumn Thermostat. Mass calibration was performed on the day of measurement using an external standard. Or on a RED-00000556 Mass Spectrometer Electrospray ionization (ESI) Thermo Orbitrap Thermo Scientific LTQ-Orbitrap. UV-*vis* absorption spectra were recorded on a Perkin-Elmer Lambda 900 spectrometer at room temperature using a 10 mm quartz cell. Photoluminescence spectra were recorded on a J&M TIDAS spectrofluorometer. Fluorescence correlation spectroscopy was performed on a LSM 880 confocal microscope (Carl Zeiss, Jena, Germany) equipped with a C-Apochromat 40×/1.2 W (Carl Zeiss, Jena, Germany) water immersion objective. The studied fluorophores were excited by a HeNe laser (633 nm) fiber coupled to the microscope.

Synthesis of DBOV-OTEG and DBOV-azide

Synthetic route towards dyes **DBOV-OTEG** (**7**) and **DBOV-Azide** (**12**) were shown in Scheme S1 and Scheme S2, respectively. Intermediates **5**[1], **9**[2] and **10**[3] were synthesized according to the literature reported procedures.

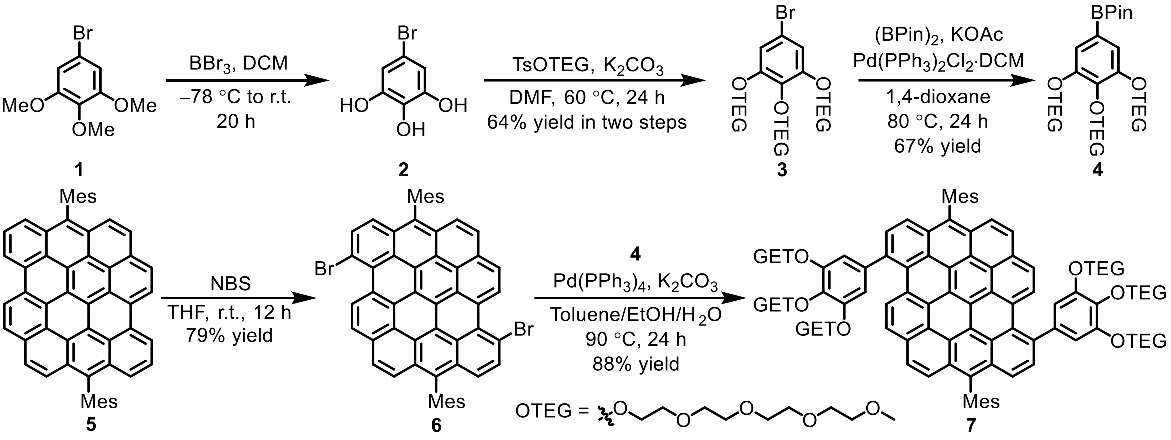

Scheme S1. Synthetic route towards **DBOV-OTEG** (**7**).

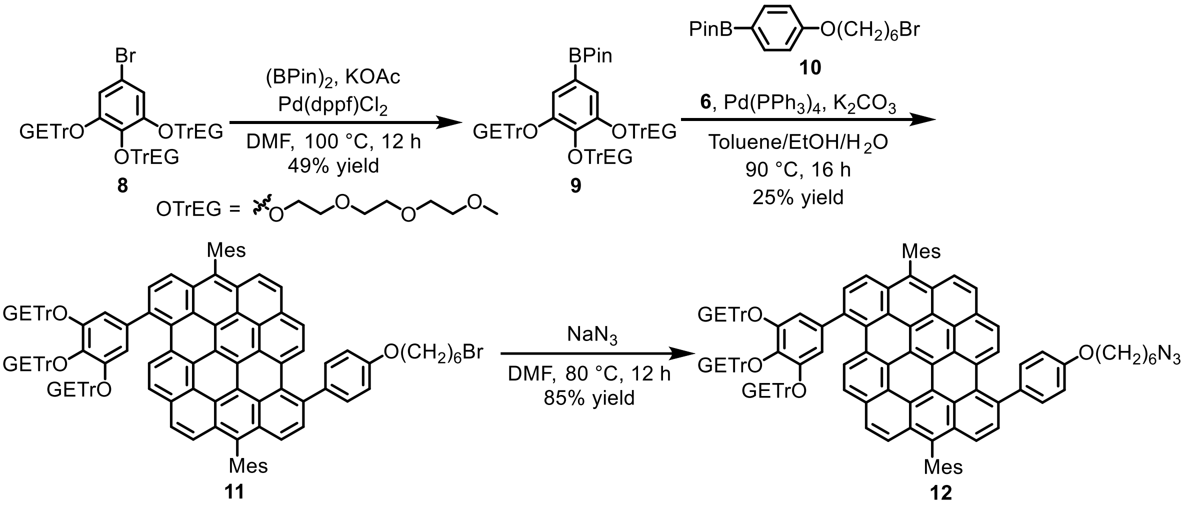

Scheme S2. Synthetic route towards **DBOV-azide** (**12**).

Synthesis of 5-bromo-1,2,3-tris(tetraethylene glycol methyl ether)benzene (**3**)

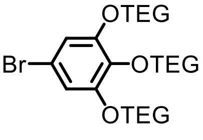

To a solution of 5-bromo-1,2,3-trimethoxylbenzene (**1**) (0.74 g, 3.0 mmol) dissolved in dry dichloromethane (10 mL) was added dropwise BBr_3 (0.96 mL, 10 mmol) at –78 °C. After addition, the reaction mixture was warmed up to room temperature and the resulting solution was stirred overnight. The mixture was poured into ice water (20 mL) and extracted with ethyl acetate (25 mL) for three times. The combined organic phase was washed with brine, dried over Na_2SO_4 and evaporated to give 5-bromo-1,2,3-trihydroxybenzene (**2**) as white solid, which was used for the next step without further purification. The residue obtained above was dissolved in dimethylformamide (DMF)

(20 mL), then K_2CO_3 and (4-toluenesulfonyl)tetraethylene glycol monomethyl ether (TsOTEG) (3.8 g, 12 mmol) were added. The resulting suspension was stirred at 60 °C for 24 hours. After cooling down to room temperature, the mixture was diluted with dichloromethane (50 mL) and washed with water (20 mL). The organic phase was separated, dried over Na_2SO_4 and evaporated to dryness under reduced pressure. The residue was purified by silica gel column chromatography (eluent: ethyl acetate to ethyl acetate/methanol = 4/1 v/v) to give the title compound (1.6 g, 64% yield over two steps) as colorless oil. ^1H NMR (300 MHz, dichloromethane-d_2) δ 6.76 (s, 2H, $\underline{H}$-Ar), 4.24 – 4.01 (m, 6H, -C$\underline{H}_2$OAr), 3.82 (t, J = 4.8 Hz, 4H, -OC$\underline{H}_2$CH$_2$OAr), 3.73 (t, J = 4.9 Hz, 2H, -OC$\underline{H}_2$CH$_2$Ar), 3.70 – 3.53 (m, 30H, -(OC$\underline{H}_2$C$\underline{H}_2$)$_2$OC$\underline{H}_2$-), 3.53 – 3.44 (m, 6H, -C$\underline{H}_2$OCH$_3$), 3.32 (s, 9H, -OC$\underline{H}_3$); ^{13}C NMR (75 MHz, dichloromethane-d_2) δ 153.88, 138.03, 116.17, 111.41, 72.89, 72.44, 72.41, 71.29, 71.10, 71.08, 71.02, 70.98, 70.92, 70.89, 70.06, 69.51, 59.17; HR MS (ESI): m/z Calcd. for $C_{33}H_{59}BrO_{15}$: 775.3110 $[M+H]^+$, found: 775.3096 (error = −1.8 ppm).

Synthesis of 3,3,4,4-tetramethyl-1-(3,4,5-tris(tetraethylene glycol methyl ether)phenyl)-borolane (**4**)

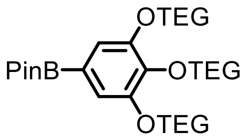

To a 25-mL Schlenk tube was added 1-bromo-3,4,5-tris(tetraethylene glycol methyl ether)benzene (**3**) (158 mg, 20.4 μmol), $Pd(PPh_3)_2Cl_2$·DCM (7 mg, 1 μmol), bis(pinacolato)diboron (76 mg, 30 μmol) and KOAc (59 mg, 60 μmol). The reaction tube was evacuated and backfilled with argon for three times, before degassed 1,4-dioxane (5 mL) was added. The solution was heated at 80 °C for 24 h. After completion of the reaction, the mixture was diluted with ether (25 mL) and washed with water (10 mL). The organic phase was washed with brine (10 mL), dried over Na_2SO_4 and evaporated under reduced pressure. The residue was purified by silica gel column chromatography (eluent: ethyl acetate/methanol = 10/1 v/v) to give the title compound (111 mg, 67% yield) as colorless oil. ^1H NMR (300 MHz, dichloromethane-d_2) δ 7.03 (s, 2H, $\underline{H}$-Ar), 4.30 – 4.13 (m, 6H, -C$\underline{H}_2$-O-Ar), 3.96 – 3.83 (m, 4H, -C$\underline{H}_2$-O-Ar), 3.81 – 3.76 (m, 2H), 3.75 – 3.57 (m, 30H), 3.57 – 3.49 (m, 6H), 3.36 (s, 9H, -OC$\underline{H}_3$), 1.34 (s, 12H, -C$\underline{H}_3$); ^{13}C NMR (75 MHz, dichloromethane-d_2) δ 152.85, 141.57, 113.76, 84.39, 72.84, 72.47, 71.28, 71.13, 71.10, 71.06, 71.01, 70.95, 70.30, 69.18, 59.18, 25.20; HR MS (ESI): m/z Calcd. for $C_{39}H_{71}BO_{17}$: 845.4677 $[M+Na]^+$, found: 845.4678 (error = 0.1 ppm).

Synthesis of **DBOV-OTEG** (**7**)

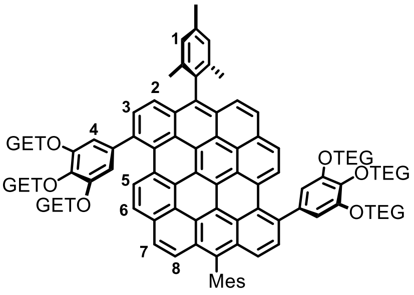

A degassed solution of 3,11-dibromo-6,14-dimesityldibenzo[*hi*,*st*]ovalene (**6**) (20 mg, 23 µmol), 3,3,4,4-tetramethyl-1-(3,4,5-tris(tetraethylene glycol methyl ether)phenyl)-borolane (**4**) (76 mg, 92 µmol), $Pd(PPh_3)_4$ (11 mg, 9.2 µmol) and K_2CO_3 (32 mg, 0.23 µmol) in a mixture of toluene/ethanol/water = 10 mL/2.5 mL/2.5 mL was heated at 90 °C for 24 h. After cooling down to room temperature, water (20 mL) was added, then the mixture was extracted with ethyl acetate (20 mL) for three times. The separated organic phases were combined, washed with brine, dried over $MgSO_4$ and evaporated. The blue residue was purified by silica gel column chromatography (eluent: ethyl acetate/methanol = 2/1 v/v) followed by size-exclusion chromatography (Bio-beads S-X3, eluent: toluene) to give the title compound (42.4 mg, 88% yield) as blue semi-solid. ^{1}H NMR (700 MHz, dichloromethane-d_2) δ 8.88 (d, J = 8.3, 2H, H-C(5)), 8.17 (d, J = 8.3 Hz, 2H, H-C(6)), 8.06 (d, J = 9.0 Hz, 2H, H-C(7)), 7.94 (s, 4H, H-C(2) and H-C(3)), 7.75 (d, J = 9.0, 2H, H-C(8)), 7.26 (s, 4H, H-C(1)), 6.99 (s, 4H, H-C(4)), 4.31 (t, J = 4.9 Hz, 4H, Ar-O-CH_2-), 4.16 – 4.12 (m, 4H Ar-O-CH_2-), 4.12 – 4.07 (m, 4H Ar-O-CH_2-), 3.90 (t, J = 4.8 Hz, 4H, -O-CH_2CH_2O-), 3.80 – 3.74 (m, 12H, -O-CH_2CH_2O-), 3.70 – 3.67 (m, 4H, -O-CH_2CH_2O-), 3.66 – 3.64 (m, 4H, -O-CH_2CH_2O-), 3.64 – 3.62 (m, 4H, -O-CH_2CH_2O-), 3.61 – 3.56 (m, 12H, -O-CH_2CH_2O-), 3.54 – 3.50 (m, 12H, -O-CH_2CH_2O-), 3.50 – 3.48 (m, 8H, -O-CH_2CH_2O-), 3.48 – 3.44 (m, 16H, -O-CH_2CH_2O-), 3.40 – 3.37 (m, 8H, -O-CH_2CH_2O-), 3.34 (s, 6H, -OCH_3), 3.24 (s, 12H, -OCH_3), 2.55 (s, 6H, Ar-CH_3), 1.96 (s, 12H, Ar-CH_3); ^{13}C NMR (175 MHz, dichloromethane-d_2) δ 154.77, 141.56, 138.57, 138.33, 138.21, 135.60, 135.40, 132.16 (C(3)), 130.68, 130.39, 129.91, 129.41, 129.31, 129.21, 129.17 (C(5) and C(1)), 129.03 (C(7)), 126.87, 126.63 (C(8)), 126.19, 125.12 (C(2)), 124.67 (C(6)), 124.35, 123.97, 123.46, 123.24, 108.75 (C(4)), 73.21, 72.49, 72.37, 71.28, 71.23, 71.16, 71.12, 71.03, 70.98, 70.95, 70.85, 70.16, 69.31, 59.19, 59.09, 21.63, 20.53; HR MS (ESI): *m/z* Calcd. for $C_{74}H_{52}O_4$: 1071.5077 $[M+2Na]^{2+}$, found: 1071.5095 (error = 1.7 ppm).

Synthesis of 3,3,4,4-tetramethyl-1-(3,4,5-tris(triethylene glycol methyl ether)phenyl)-borolane (**9**)

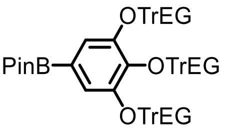

To a Schlenk tube equipped with a stirring bar was added **8** (500 mg, 0.779 mmol), bis(pinacolato)diboron (508 mg, 2.00 mmol), Pd(dppf)Cl_2 (90.0 mg, 0.123 mmol) and potassium acetate (245 mg, 12.5 mmol). The reaction tube was evacuated and backfilled

with argon for three times before the addition of DMF (20 mL). The mixture was degassed by bubbling with argon for 30 min and heated at 90 °C for 12 h. After cooling down to room temperature, water was added and the mixture was extracted with ethyl acetate for 3 times. The organic phases were combined, washed with brine, dried over $MgSO_4$ and evaporated. The residue was purified by silica gel column chromatography (eluent: ethyl acetate/methanol = 4/1 v/v) to give the title compound (263 mg, 49% yield) as brown oil. ^{1}H NMR (500 MHz, chloroform-*d*, 298 K) δ 7.02 (s, 2H, $\underline{H}$-Ar), 4.20 – 4.16 (m, 6H, Ar-OC$\underline{H}_2$-), 3.85 (t, J = 4.9 Hz, 4H, Ar-OCH$_2$C$\underline{H}_2$O-), 3.78 (t, J = 4.9 Hz, 2H, Ar-OCH$_2$C$\underline{H}_2$O-), 3.74 – 3.70 (m, 6H, Ar-OCH$_2$CH$_2$O-C$\underline{H}_2$), 3.67 – 3.62 (m, 12H, -C$\underline{H}_2$OC$\underline{H}_2$CH$_2$OMe), 3.55 – 3.52 (m, 6H, -C$\underline{H}_2$OMe), 3.37 (s, 9H, -OC$\underline{H}_3$), 1.32 (s, 12H, -C$\underline{H}_3$ (BPin)). ^{13}C NMR (125 MHz, chloroform-*d*, 298 K) δ (ppm) 152.35, 141.11, 113.67, 83.98, 72.33, 72.03, 70.87, 70.80, 70.75, 70.66, 70.62, 70.58, 69.83, 68.73, 59.17, 24.96. HR MS (ESI): *m/z* Calcd. for $C_{33}H_{59}BO_{14}$: 690.3992 $[M]^+$, found: 690.3973 (error = –2.8 ppm).

Synthesis of **DBOV-Mes-OTrEG-Br 11**

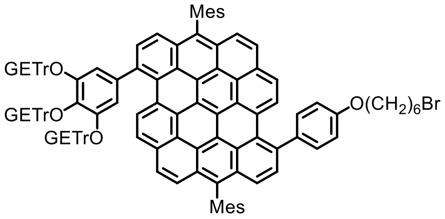

To a Schlenk tube equipped with a stirring bar was added **6** (14.0 mg, 16.2 μmol), **9** (12.1 mg, 17.5 μmol), **10** (6.70 mg, 17.5 μmol), $Pd(PPh_3)_4$ (3.00 mg, 2.60 μmol) and K_2CO_3 (13.8 mg, 100 μmol). The reaction tube was evacuated and backfilled with argon for three times before a mixture of toluene/ethanol/water = 4 mL/1 mL/1 mL was added. The mixture was degassed by three freeze-pump-thaw cycles and heated at 90 °C for 16 h. After cooling down to room temperature, the reaction mixture was extracted with ethyl acetate for 3 times. The organic layers were combined, washed with brine, dried over $MgSO_4$ and evaporated. The residue was purified by silica gel column chromatography (eluent: ethyl acetate/methanol = 20/1 v/v) to give the title compound (6.10 mg, 25% yield) as blue oil. ^{1}H NMR (400 MHz, chloroform-*d*, 298 K) δ (ppm) 8.86 (d, J = 8.4 Hz, 1H), 8.79 (d, J = 8.4 Hz, 1H), 8.08 (dd, J = 8.6, 2.2 Hz, 2H), 7.99 (d, J = 9.2 Hz, 2H), 7.95 – 7.91 (m, 2H), 7.89 – 7.84 (m, 2H), 7.74 (dd, J = 9.1, 3.1 Hz, 2H), 7.64 (d, J = 8.6 Hz, 2H), 7.24 (s, 4H), 7.08 (d, J = 8.7 Hz, 2H), 6.95 (s, 2H), 4.33 (t, J = 5.1 Hz, 2H), 4.16 – 4.09 (m, 6H), 3.92 (t, J = 5.1 Hz, 2H), 3.84 – 3.80 (m, 4H), 3.75 – 3.68 (m, 6H), 3.66 – 3.56 (m, 12H), 3.55 – 3.51 (m, 6H), 3.47 (t, J = 6.68 Hz, 2H), 3.39 (s, 9H), 2.55 (s, 6H), 1.97 (d, J = 4.0 Hz, 12H), 1.92 – 1.87 (m, 4H), 1.37 – 1.32 (m, 4H); HR MS (ESI): *m/z* Calcd. for $C_{95}H_{101}BrNO_{14}$:1542.6451 $[M+NH_4]^+$, found:1542.6405 (error = –3.0 ppm).

Synthesis of **DBOV-azide 12**

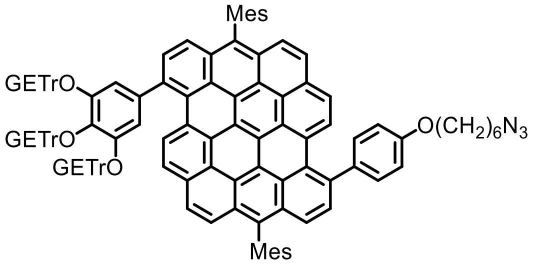

To a Schlenk tube equipped with a stirring bar was added **11** (1.2 mg, 0.79 μmol) and NaN_3 (20.0 mg, 307 μmol). The reaction tube was evacuated and backfilled with argon for three times before the addition of DMF (2 mL). The mixture was degassed by three freeze-pump-thaw cycles and heated at 80 °C for 12 h. After cooling down to room temperature, the reaction mixture was diluted with ethyl acetate, washed with water, brine, dried over Na_2SO_4 and evaporated. The residue was purified by silica gel column chromatography (eluent: ethyl acetate/methanol = 20/1 v/v) to give the product (1.00 mg, 85% yield) as blue oil. ^{1}H NMR (500 MHz, chloroform-*d*, 298 K) δ (ppm) 8.86 (d, J = 8.4 Hz, 1H), 8.78 (d, J = 8.4 Hz, 1H), 8.08 (dd, J = 8.5, 3.6 Hz, 2H), 7.99 (d, J = 9.2 Hz, 2H), 7.95 – 7.91 (m, 2H), 7.89 – 7.84 (m, 2H), 7.74 (dd, J = 9.1, 3.5 Hz, 2H), 7.64 (d, J = 8.1 Hz, 2H), 7.24 (s, 4H), 7.08 (d, J = 8.3 Hz, 2H), 6.94 (s, 2H), 4.32 (t, J = 5.2 Hz, 2H), 4.16 – 4.09 (m, 6H), 3.92 (t, J = 5.2 Hz, 2H), 3.84 – 3.80 (m, 4H), 3.75 – 3.68 (m, 6H), 3.66 – 3.56 (m, 12H), 3.55 – 3.51 (m, 6H), 3.39 (s, 9H), 3.33 (t, J = 6.69 Hz, 2H), 2.55 (s, 6H), 1.96 (d, J = 5.2 Hz, 12H), 1.92 – 1.85 (m, 4H), 1.37 – 1.34 (m, 4H); HR MS (ESI): *m/z* Calcd. for $C_{95}H_{101}N_4O_{13}$: 1505.7360 $[M+NH_4]^+$, found:1505.7304 (error = –3.7 ppm).

Sample preparation for single-molecule measurements

Coverslip cleaning and coating of polystyrene film were done according to the previous literature[4,5]. In brief, the coverslips were sonicated in 1% Micro 90 alkaline cleaning solution for 15 min. Then the coverslips were rinsed three times with Milli-Q water and finally dried with nitrogen flow. Afterward, those coverslips were cleaned by oxygen-plasma cleaner (250 W, 10 min).

The purification of polystyrene was carried out using an anti-solvent crystallization method. The polystyrene solid was dissolved in anhydrous THF to obtain a clear solution. Then an equal volume of MeOH was added to the solution, and the resulting mixture was let stand until it became a clear solution again, and all the crystals were precipitated. The solvents were subsequently removed, and the precipitates were washed with MeOH, and then dried in a vacuum desiccator prior to the use. 100 μL of the solution of polystyrene purified above (4 mg/mL in toluene) was spin-coated on the cleaned coverslip. The coverslip was first spun at 2,000 rpm for 20 s and then at 4,000 rpm for 40 s. The sample was dried on a hot plate by heating at 90 °C for 10 min.

Preparation of nanographene samples for single-molecule blinking property measurements in PBS and different pH values solutions

10 μL of a solution of nanographene (10^{-12} M in toluene/ethanol=1/99) was spin-coated on the polystyrene-coated coverslip. The coverslip was firstly spun at 2,000 rpm for 20 s and then at 4,000 rpm for 40 s. The sample was dried on a hot plate by heating at 70 °C for 15 min.

Preparation of nanographene samples for single-molecule blinking property measurements in air

10 μL of a solution of nanographene (10^{-12} M in toluene) was spin-coated on the cleaned coverslip. The coverslip was first spun at 2,000 rpm for 20 s and then at 4,000 rpm for 40 s. The sample was dried on a hot plate by heating at 70 °C for 15 min.

Preparation of Aβ fiber

Aβ (No. RP10017) was purchased from Genscript as Aβ1–42 "click peptide". These click peptides can be easily converted to native peptide at pH 7.4 or above. Aβ1–42 peptide received was diluted with DPBS to a final concentration of 100 μM and stored in a -80 ºC freezer until use. This peptide solution is a mixture of monomers and oligomers without pre-monomerization. For fibrils formation, 80 μL of DBOV-OTEG (10 nM) in DMSO was added to 20 μL Aβ1-42 peptide solution, then was incubated for five days at 37 °C. After Aβ fibrils formation, ThT solution was added at a final concentration of 200 nM for 30 min. Subsequently, 10 μL of the above solution was dropped on a clean circular coverglass (#1.5, 170 μm thickness) and dried in a vacuum desiccator. The fiber-deposited coverglass was washed three times with water to remove excess salt and unfixed fibers and then dried again for further measurement.

Live cell SMLM imaging of lysosomes with DBOV-OTEG

U2OS cells were cultured in Dulbecco's Modified Eagle's Medium (DMEM) supplemented with 10% fetal bovine serum (FBS), in a 5% CO_2 humidified incubator at 37 °C. The cells were then plated into 35 mm diameter glass bottom Petri dishes (ibidi) and incubated overnight under the same condition. Live U2OS cells were stained for four hours with 1 μM DBOV-OTEG in DMEM (supplement 10% FBS) medium, rinsed three times in phenol red-free DMEM (each time for five minutes), and 75 nM LysoTracker Green in DMEM was added for 30 minutes, and then washed twice with DMEM, finally replaced the washing medium with DMEM (supplement 10% FBS) before imaging.

Preparation of neurons sample and imaging

DRG neurons were incubated with 10 μM o-propargyl-puromycin (OPP - custom synthesized) diluted in the cell culture medium for 15 min at 37 °C. Neurons were then washed three times with PBS and then fixed for 20 min with 4% PFA in PBS at room temperature. Neurons were permeabilized for 15 min with PBS + Triton X-100 (0.3% w/v) and washed three times with regular PBS. Neurons were then incubated for 2 h at room temperature with the click mix solution: PBS at pH 7.8, 2 mM TBTA (Tris((1-benzyl-4-triazolyl)methyl)amine), 1 mM TCEP (tris(2-carboxyethyl)phosphine), 2 uM DBOV, 0.5 mM CuSO4. Neurons were washed three times for 10 min with the click wash buffer: 0.5 mM EDTA, 1% Tween 20, PBS pH 7.8, and washed with regular PBS twice. DAPI in PBS staining was done for 20 min followed by two washes with regular PBS, and then mounting coverslips in PBS sealed with nail polish.

Control experiment 1: As a control, neurons were pre-incubated with 40 μM anisomycin (Sigma, A9789) for 30 min followed by 10 μM o-propargyl-puromycin (OPP - custom synthesized) diluted in the cell culture medium for 15 min at 37 °C. Cells were then washed three times with PBS and then fixed for 20 min with 4% PFA in PBS at room temperature, and then subjected to the same click reaction using DBOV-azide.

Control experiment 2: Neurons were incubated in the cell culture medium for 15 min at 37 °C. Cells were washed three times with PBS and fixed for 20 min with 4% PFA in PBS at room temperature, and then subjected to the same click reaction using DBOV-azide.

Measurement of single-molecule blinking characterization and super-resolution imaging

Both single-molecule blinking property measurement and super-resolution imaging were performed using the SR GSD microscope (Leica). 642 nm (500 mW) and 488 nm (500 mW) laser were selected for fluorescence reactivation. For the 642 nm laser, the excitation filter (637–647 nm/400–410 nm), the dichroic beam splitter (637–647 nm/400–410 nm), and the emission filter (660–760 nm/449–451 nm) were used. For the 488 nm laser, the excitation filter (483–493 nm/400–410 nm), the dichroic beam splitter (483–493 nm/400–410 nm), and the emission filter (500–550 nm/449–451 nm) were used. The objective lens HCX PL APO 160×1.43 NA Oil CORR-TIRF was selected for single-molecule measurements and super-resolution imaging. The microscope was equipped with an EMCCD camera (iXonDU-897, Andor). The camera settings were 10 MHz at 14 bit and a pre-amplification of 5.1. Please note here that the double bandwidth of the filters/beam splitter were chosen for 405 nm back pumping and in our experiments mentioned in this work, such back pumping was not used. For single-molecule blinking measurement, 20,000 frames were recorded with an exposure time of 30 ms, EM gain of 100, 642 nm laser power of 5 kW/cm^2. For super-resolution imaging of amyloid fibrils labeled with DBOV-OTEG in air and various pH value

solutions, 20,000 frames were recorded with an exposure time of 50 ms, EM gain of 100, 642 nm laser power of 5 kW/cm^2. We note here that at 642 nm, the absorption cross-section of DBOV-OTEG in water is around 3.57 x 10^{-3} nm^2. For live-cell super-resolution imaging, 6,500 frames were recorded with an exposure time of 23 ms, EM gain of 100, 642 nm laser power of 1 kW/cm^2. For super-resolution imaging of global nascent proteins labeled with DBOV-azide in PBS, 40,000 frames were recorded with an exposure time of 30 ms, EM gain of 100, 642 nm laser power of 5 kW/cm^2.

SMLM image data analysis

All SMLM movies were analyzed with ThunderSTORM Plugin in ImageJ[6]. The peak intensity threshold was set as 1.7. And sigma > 90 and sigma < 150 were used to collect the true signal-molecule signal with a wavelength of 660 to 760 nm. Drift correction was done with cross-correlation function (number of bins of 3) in ThunderSTORM Plugin in ImageJ. For signal-molecule blinking characterization and super-resolution imaging of neurons, the continuous fluorescence signal was merged as one molecule. The localization precision of single-molecule was calculated based on Maximum-Likelihood methods[7] with ThunderSTORM Plugin in ImageJ[6]. All super-resolution images were reconstructed in ThunderSTORM, for SMLM image of amyloid fibrils - pixel size of 10 nm, for SMLM images of lysosomes and neurons - pixel size of 5 nm. The localizations of global nascent proteins were used for Voronoi analysis (software: SR-Tesseler[8]). All parameters are default (Density factor: 1, Min area: 2, Min # of locs: 5, Max are: 10,000, Max # of locs: 100,000).

NMR spectra

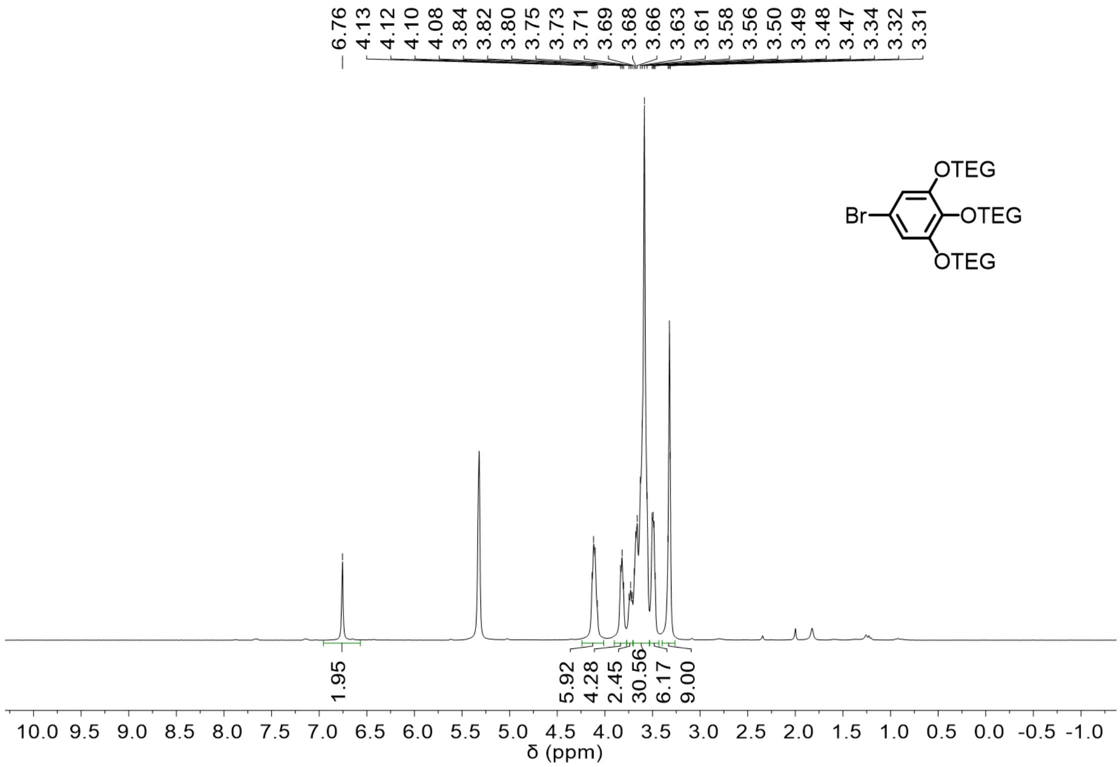

Figure S1. ^{1}H NMR spectrum of 5-bromo-1,2,3-tris(tetraethylene glycol methyl ether)benzene (**3**) (300 MHz, dichloromethane-d_2, 298 K).

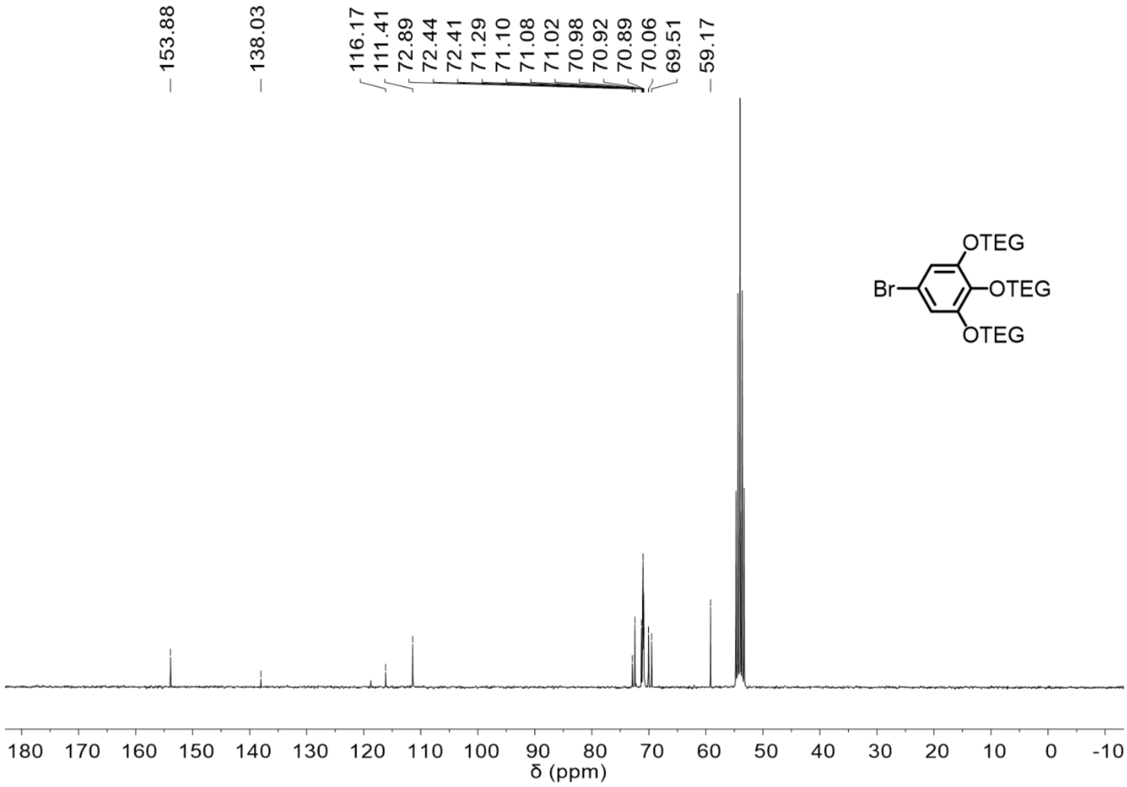

Figure S2. ^{13}C NMR spectrum of 5-bromo-1,2,3-tris(tetraethylene glycol methyl

ether)benzene (**3**) (75 MHz, dichloromethane-d_2, 298 K)

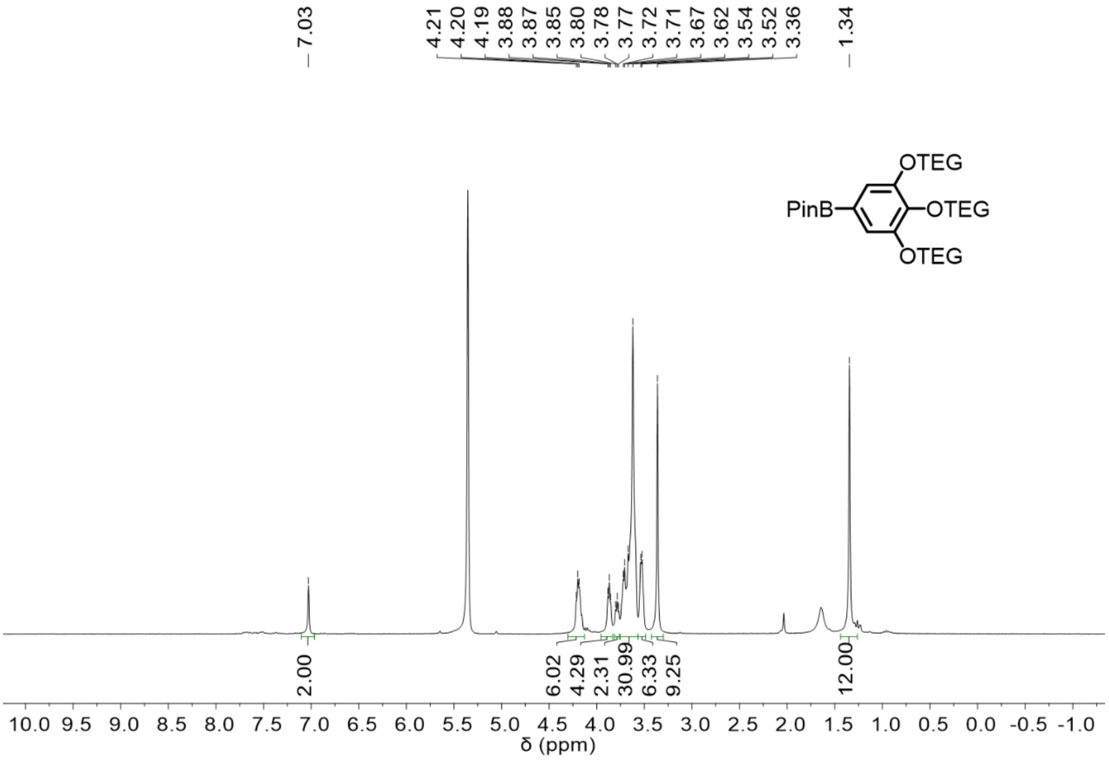

Figure S3. ^{1}H NMR spectrum of 3,3,4,4-tetramethyl-1-(3,4,5-tris(tetraethylene glycol methyl ether)phenyl)-borolane (**4**) (300 MHz, dichloromethane-d_2, 298 K).

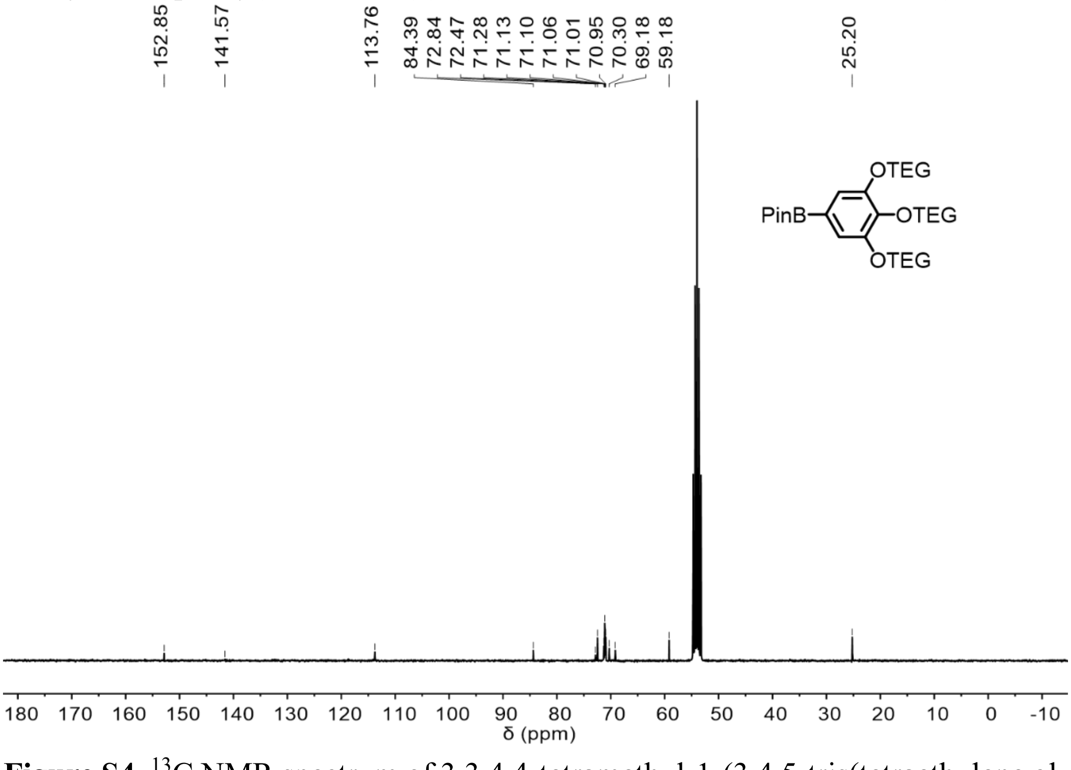

Figure S4. ^{13}C NMR spectrum of 3,3,4,4-tetramethyl-1-(3,4,5-tris(tetraethylene glycol methyl ether)phenyl)-borolane (**4**) (75 MHz, dichloromethane-d_2, 298 K).

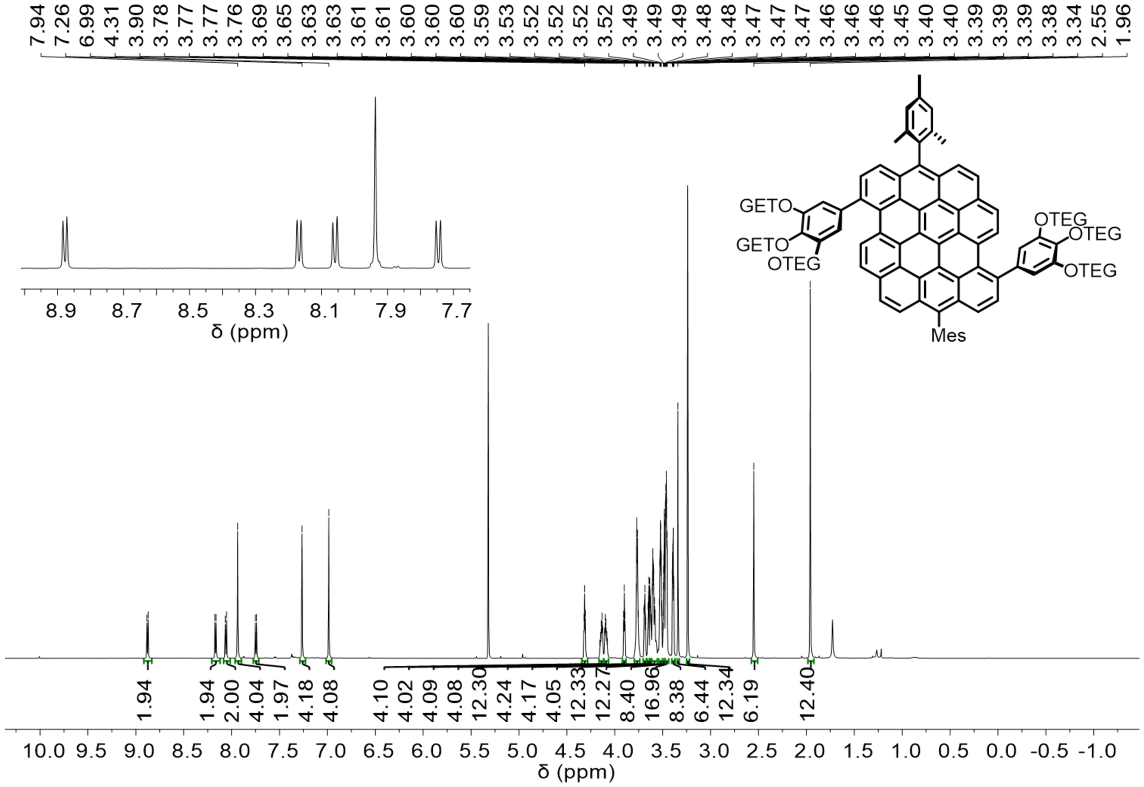

Figure S5. ^{1}H NMR spectrum of DBOV-Mes-OTEG (**7**) (700 MHz, dichloromethane-d_2, 298 K).

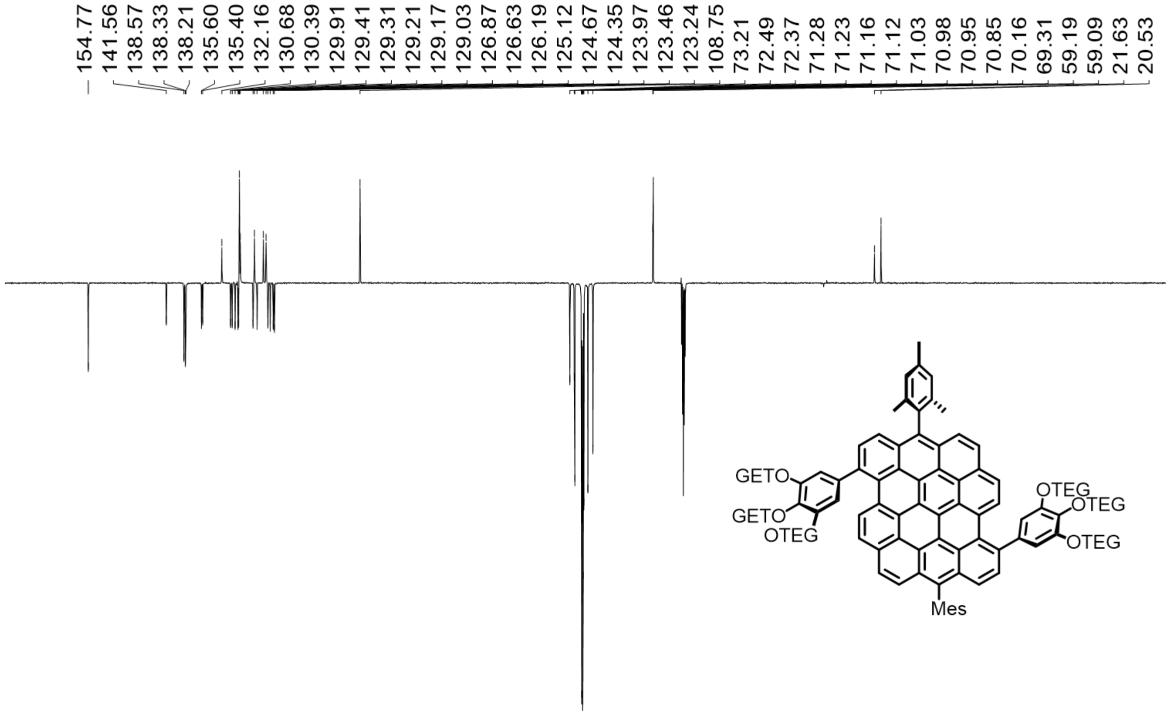

Figure S6. APT ^{13}C NMR spectrum of DBOV-OTEG (**7**) (175 MHz, dichloromethane-d_2, 298 K).

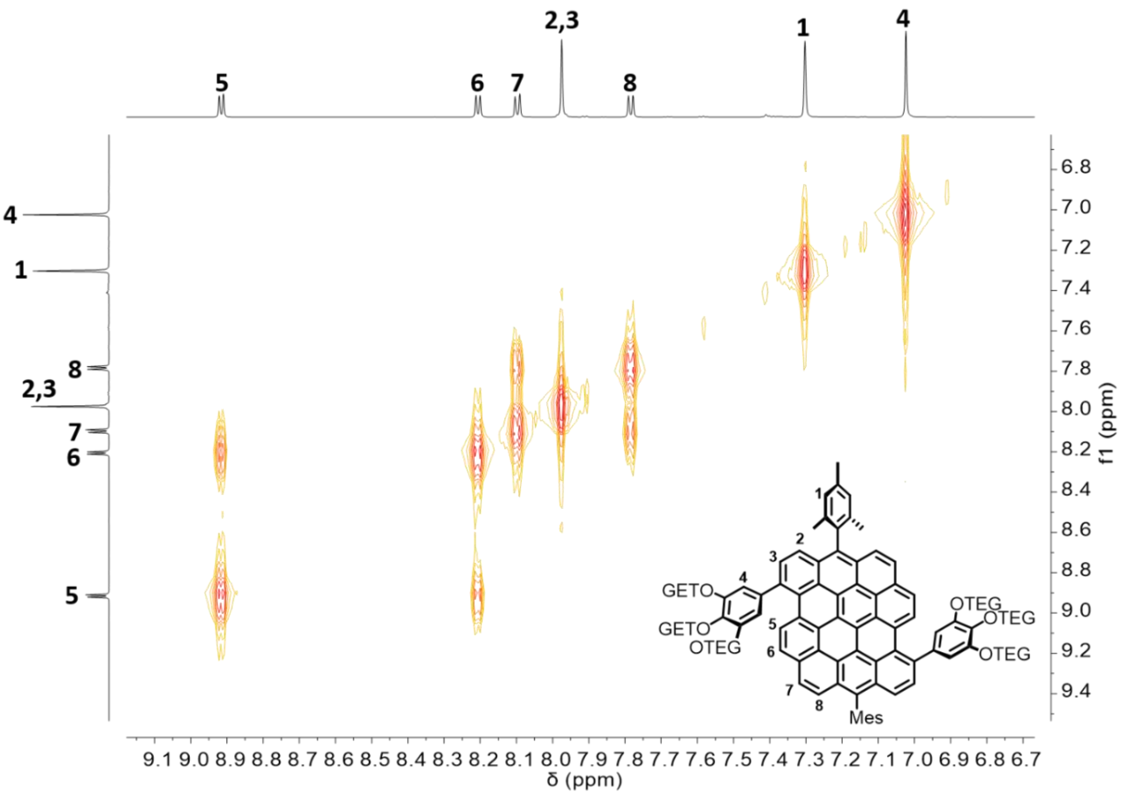

Figure S7. Aromatic region of ^{1}H,^{1}H-COSY spectrum of DBOV-OTEG (**7**) (700 MHz, dichloromethane-d_2, 298 K).

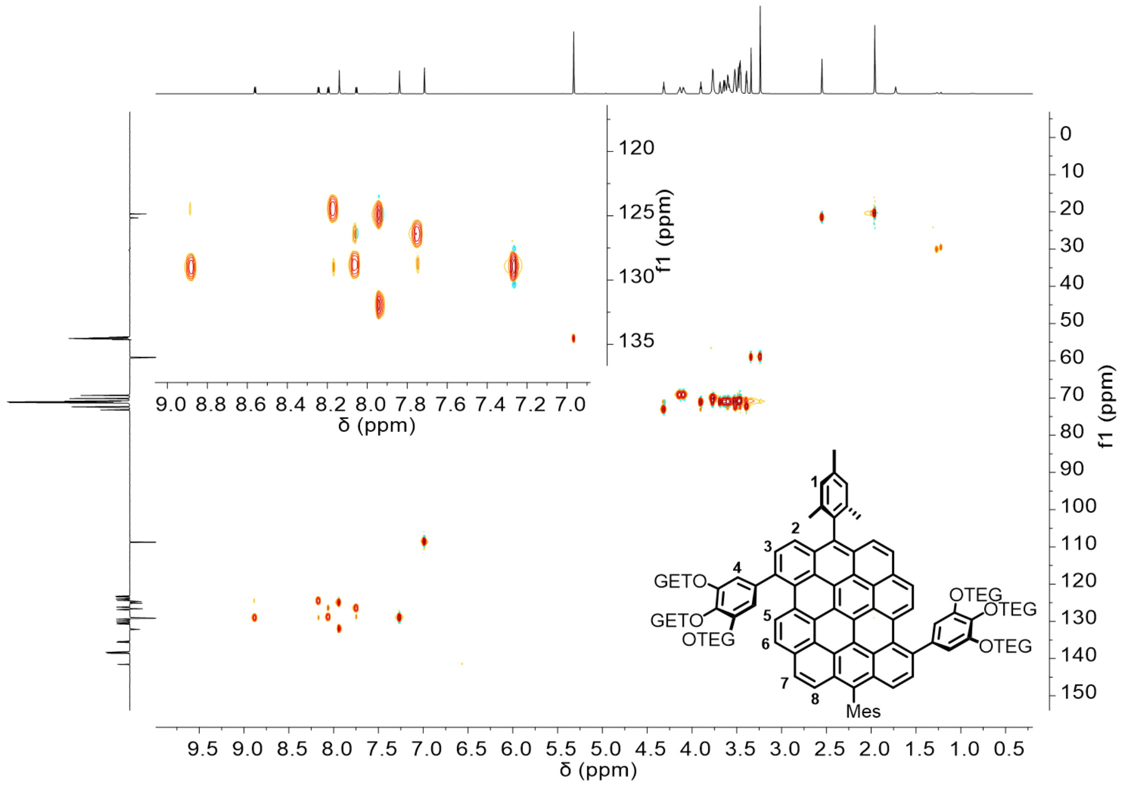

Figure S8. ^{1}H, ^{13}C-HSQC spectrum of DBOV-OTEG (**7**) (700 MHz, dichloromethane-d_2, 298 K).

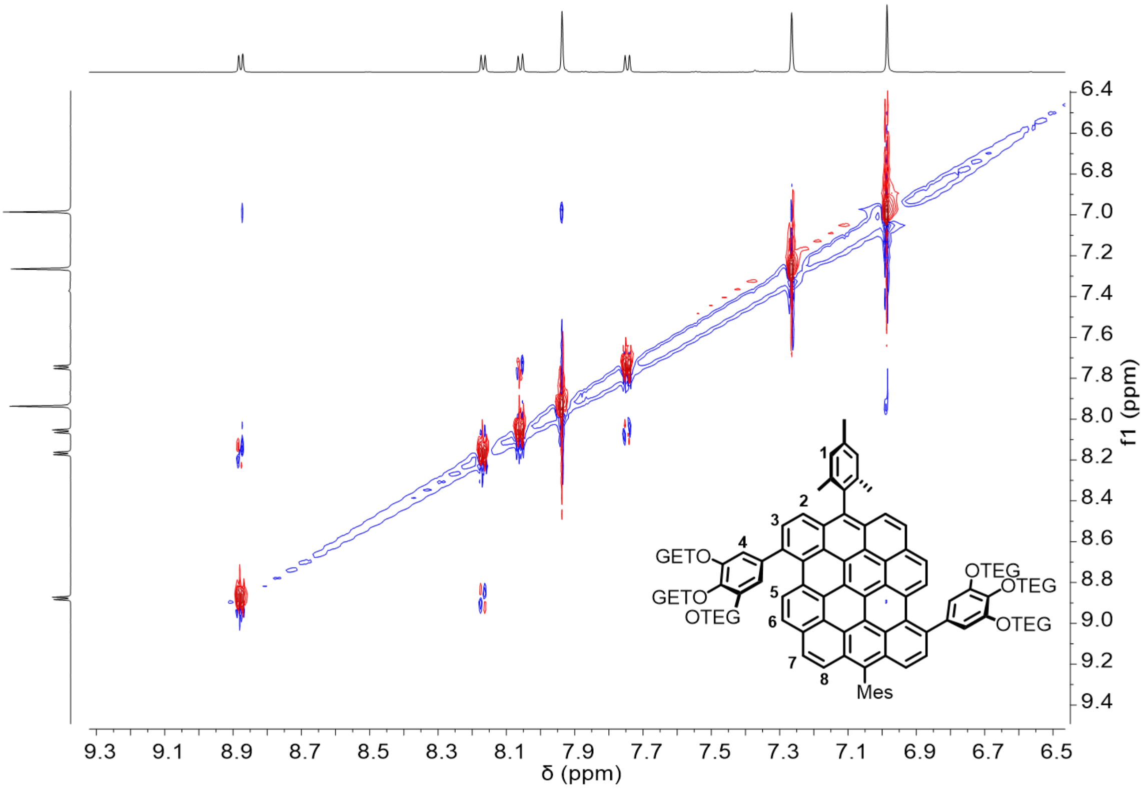

Figure S9. Aromatic region of ^{1}H,^{1}H-NOESY spectrum of DBOV-OTEG (**7**) (700 MHz, dichloromethane-d_2, 298 K).

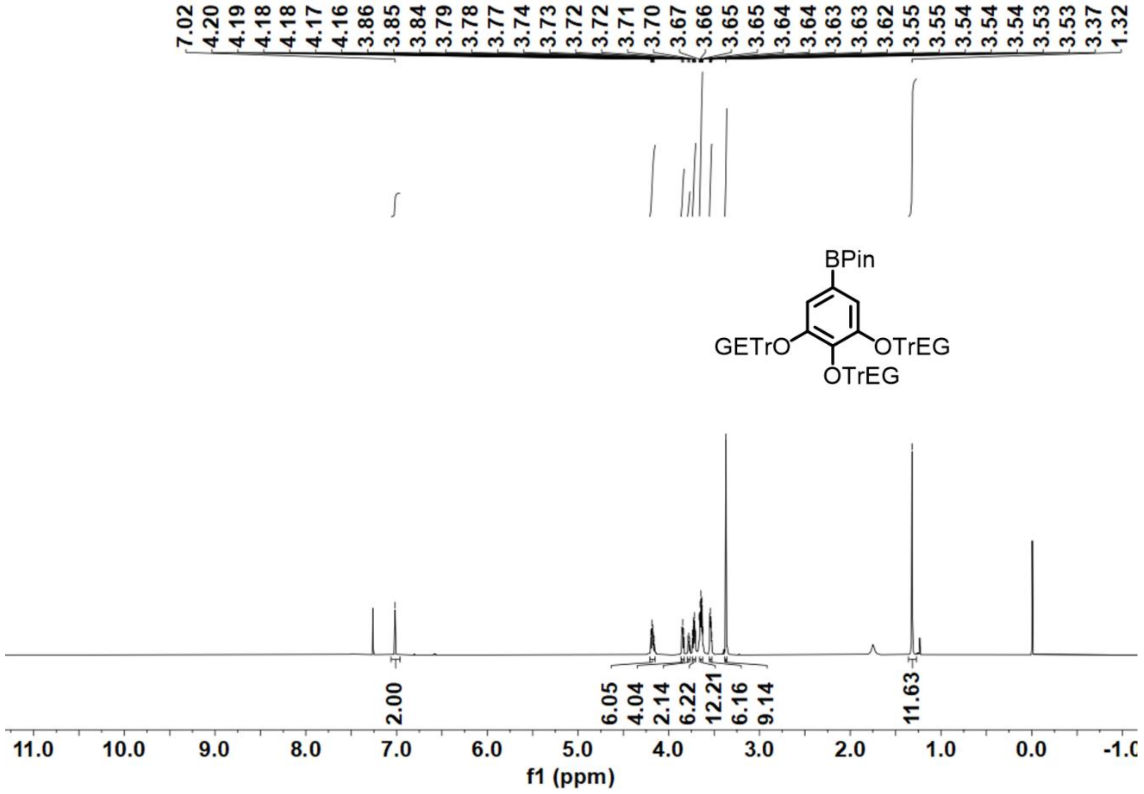

Figure S10. ^{1}H NMR spectrum of 3,3,4,4-tetramethyl-1-(3,4,5-tris(triethylene glycol methyl ether)phenyl)-borolane (**9**) (500 MHz, chloroform-d, 298K).

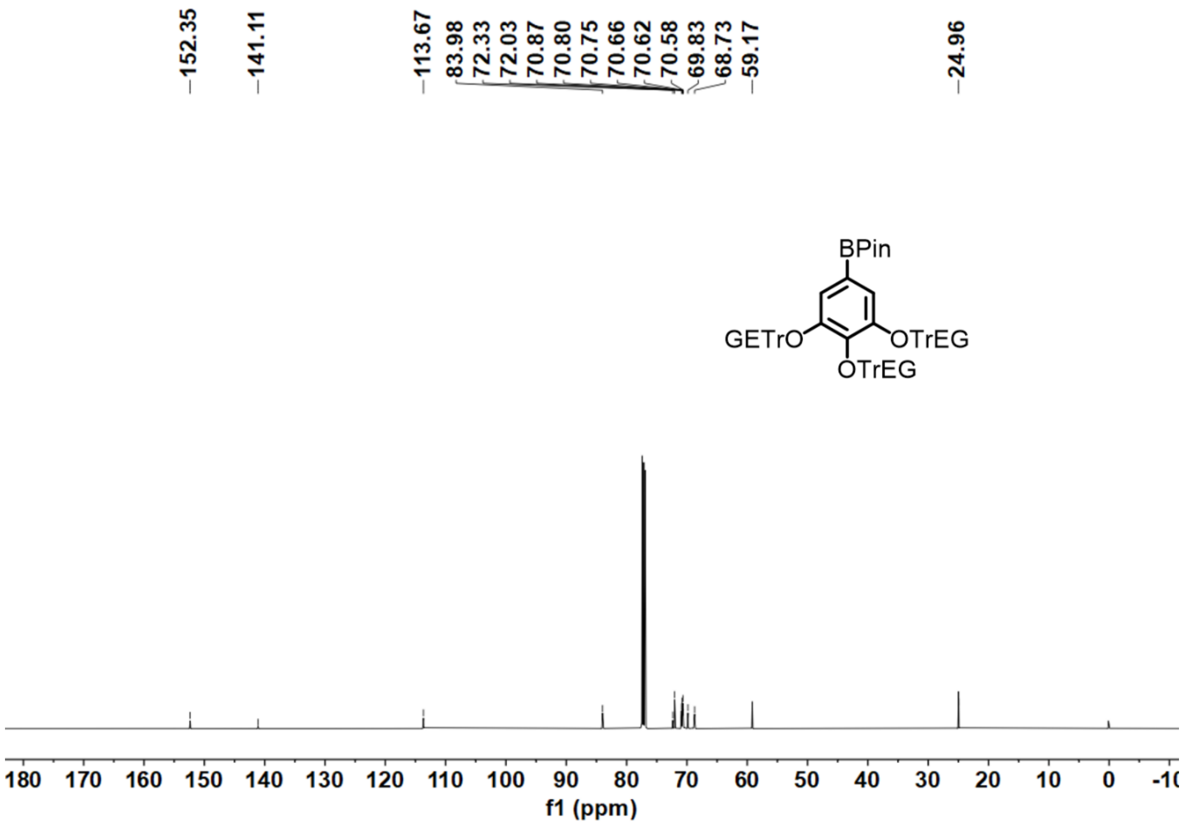

Figure S11. ^{13}C NMR spectrum of 3,3,4,4-tetramethyl-1-(3,4,5-tris(triethylene glycol methyl ether)phenyl)-borolane (**9**) (500 MHz, chloroform-*d*, 298K).

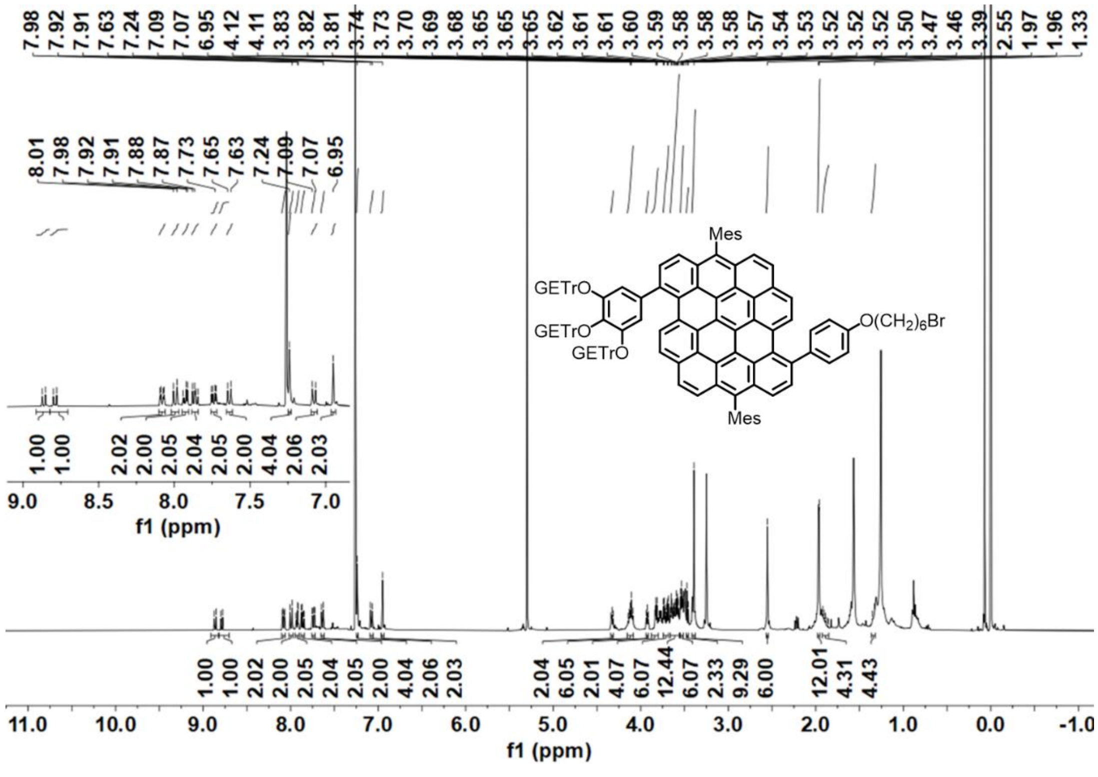

Figure S12. ^{1}H NMR spectrum of **11** (400 MHz, chloroform-*d*, 298K).

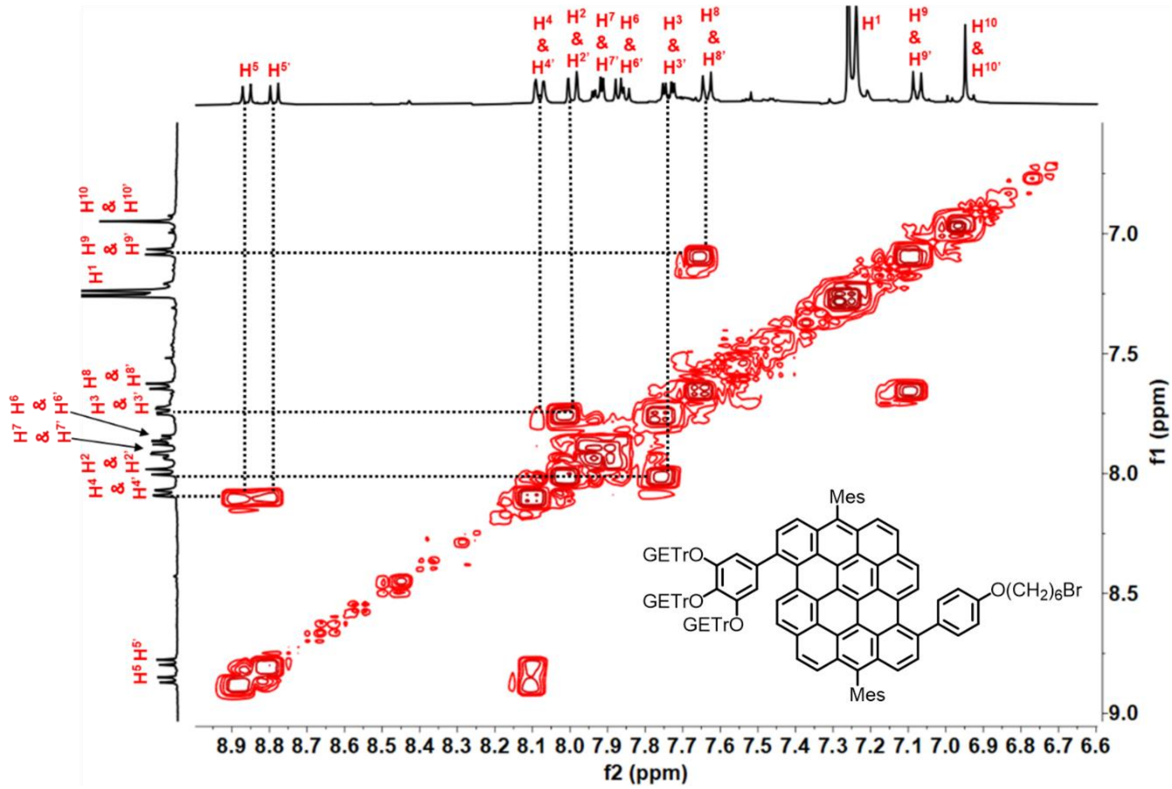

Figure S13. Aromatic region of ^{1}H-^{1}H COSY spectrum of **11** (400 MHz, chloroform-*d*, 298K).

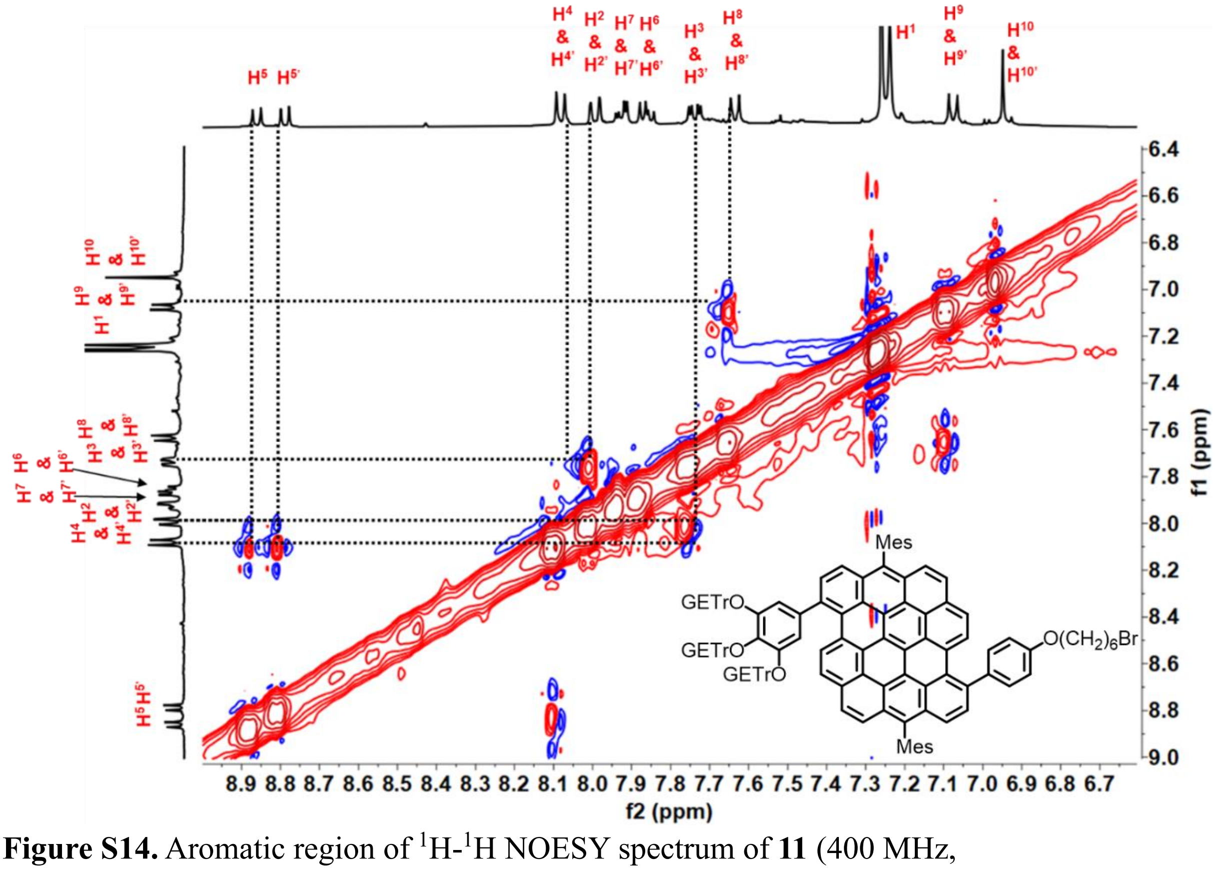

Figure S14. Aromatic region of ^{1}H-^{1}H NOESY spectrum of **11** (400 MHz, chloroform-*d*, 298K).

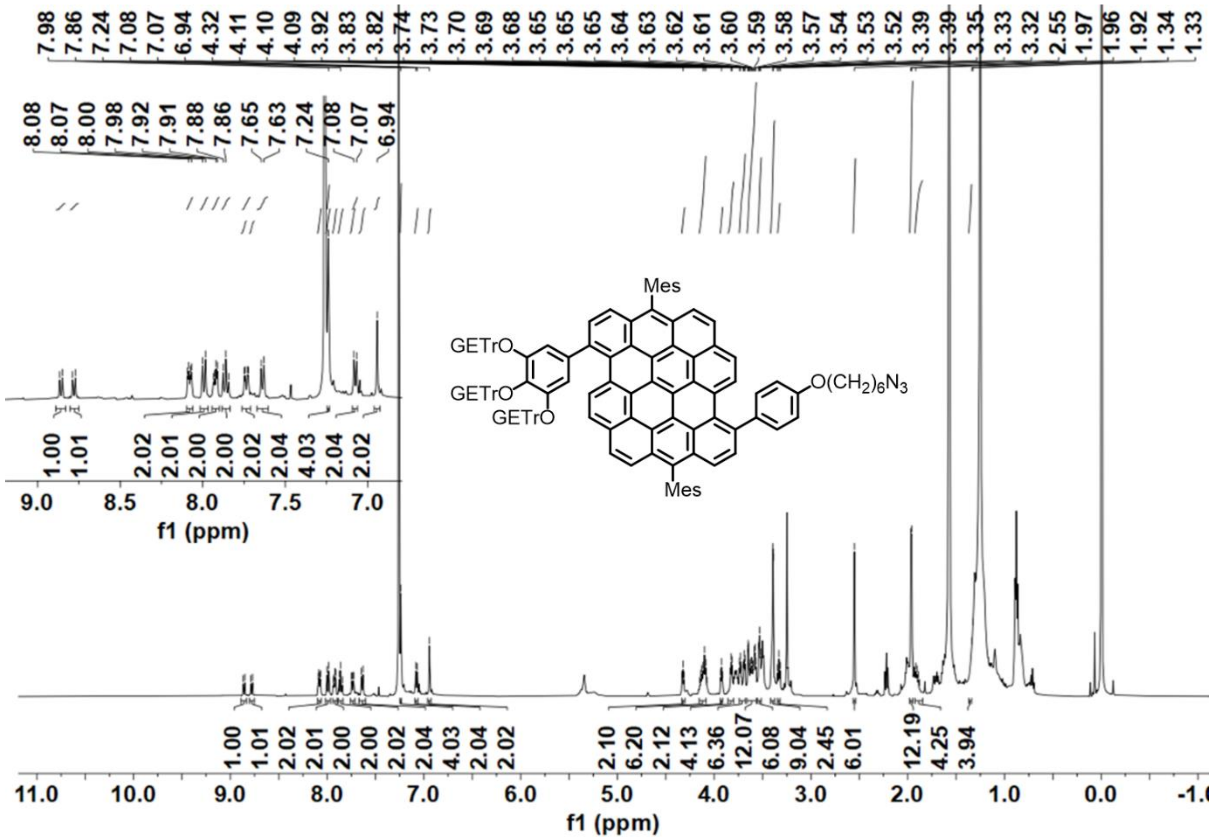

Figure S15. ^{1}H NMR spectrum of DBOV-azide **12** (500 MHz, chloroform-*d*, 298K).

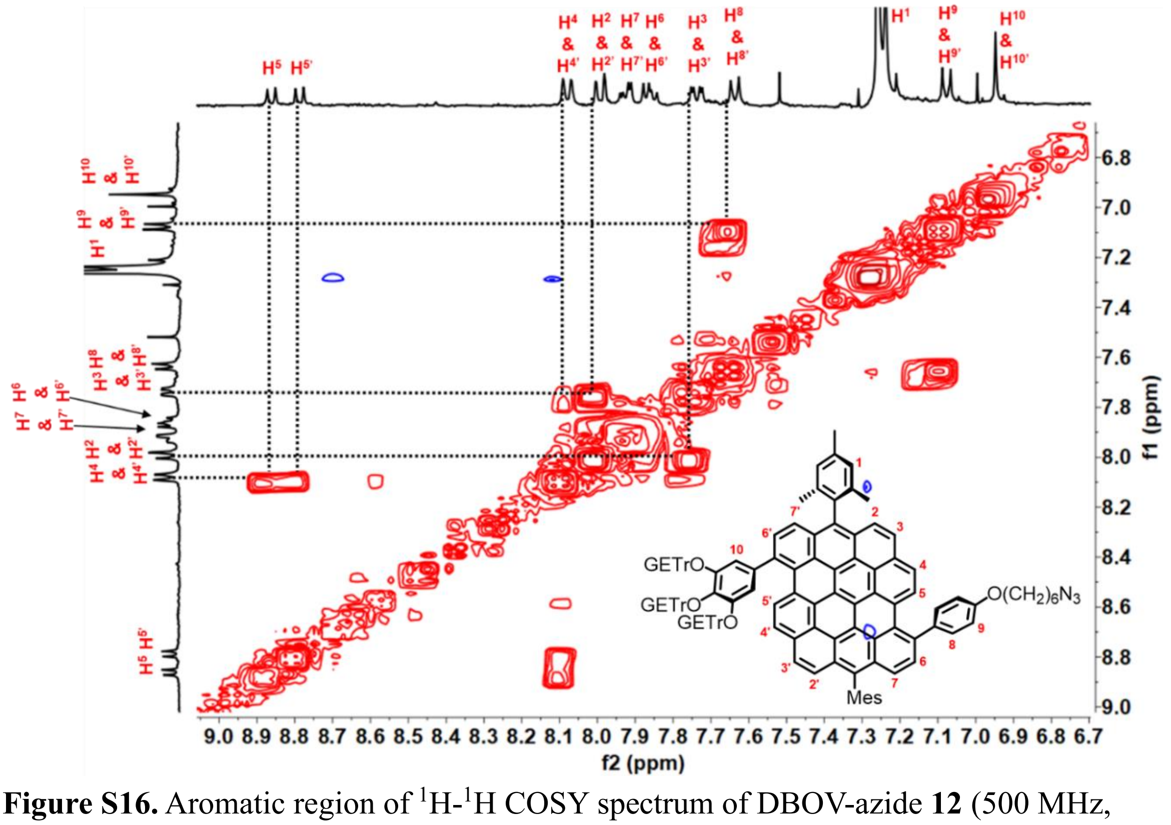

Figure S16. Aromatic region of ^{1}H-^{1}H COSY spectrum of DBOV-azide **12** (500 MHz, chloroform-*d*, 298K).

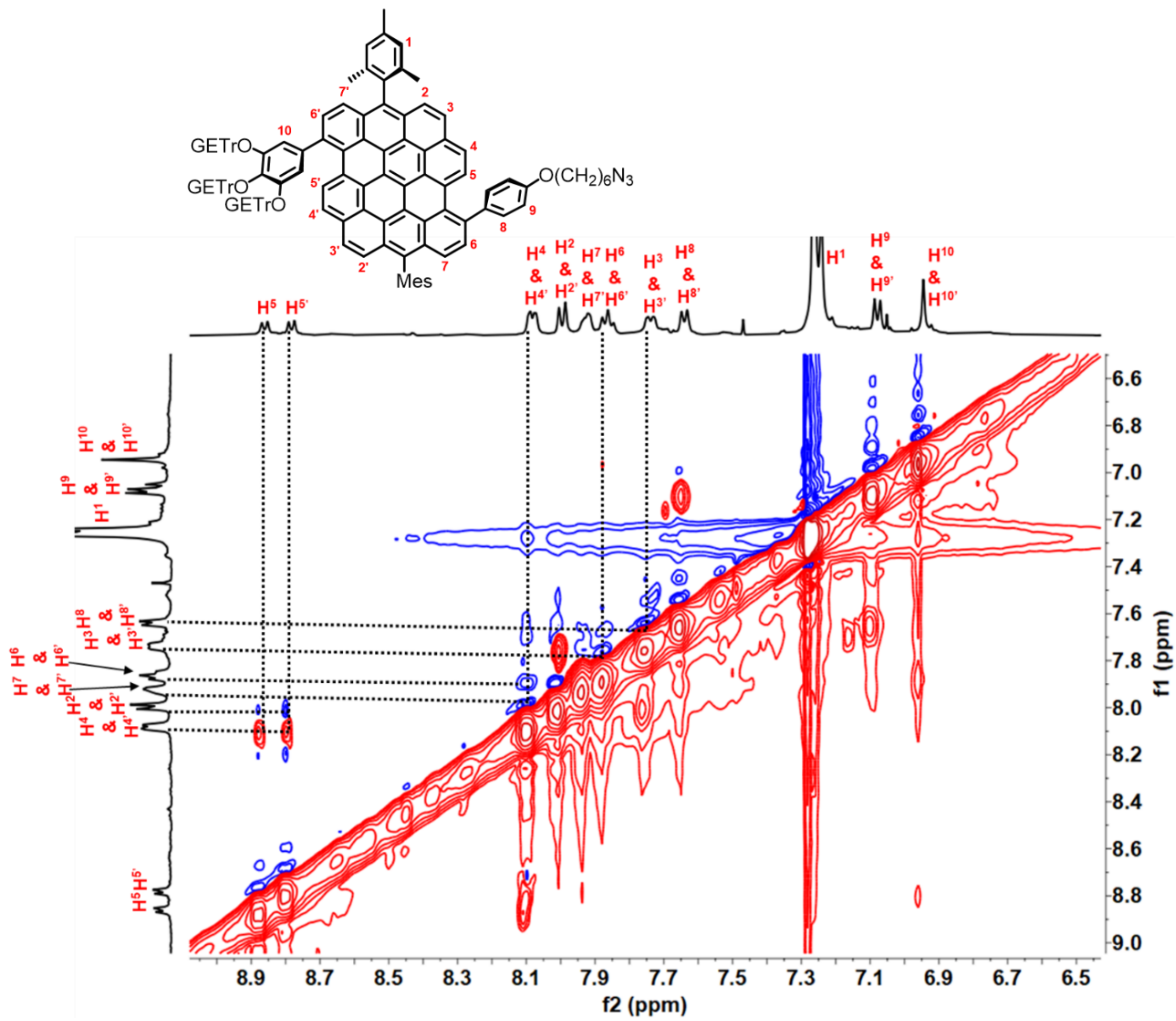

Figure S17. Aromatic region of ^{1}H-^{1}H NOESY spectrum of DBOV-azide **12** (500 MHz, chloroform-*d*, 298K).

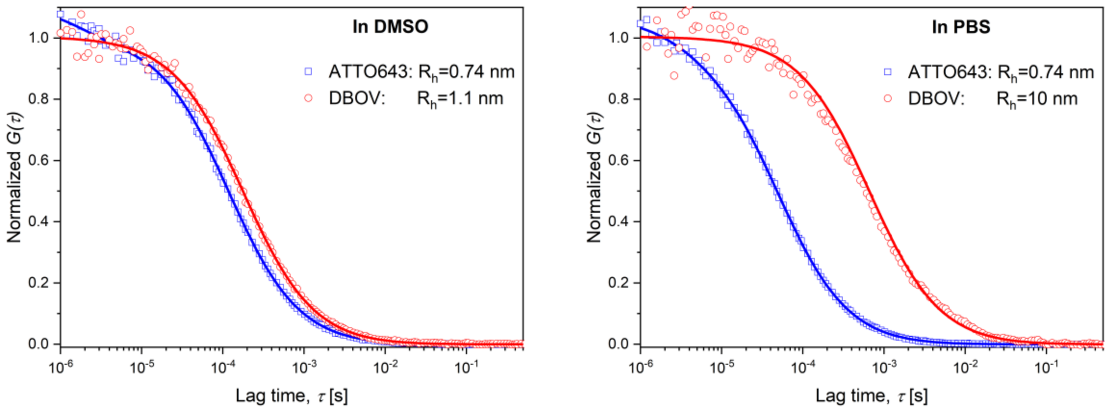

Figure S18. Characterization of the DBOV-OTEG solubility by fluorescence correlation spectroscopy (FCS). Normalized FCS autocorrelation curve for DBOV-OTEG (red circles) and a reference dye ATTO643 (blue squares) measured in DMSO (left panel) and in PBS (right panel). In DMSO, the hydrodynamic radius (R_h) of DBOV-OTEG is around 1 nm indicating very good solubility. In PBS, the R_h of DBOV-OTEG is around 10 nm indicating the formation of very small aggregates.

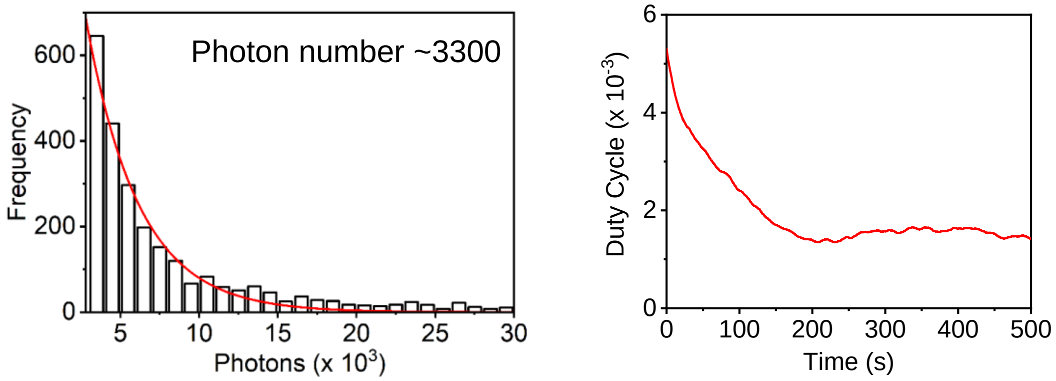

Figure S19. Blinking properties of single molecule DBOV-OTEG in air, Left: Histogram of detected photons per switching event and single-exponential fit. Right: On-off duty cycle (fraction of time a molecule resides in its fluorescent state).

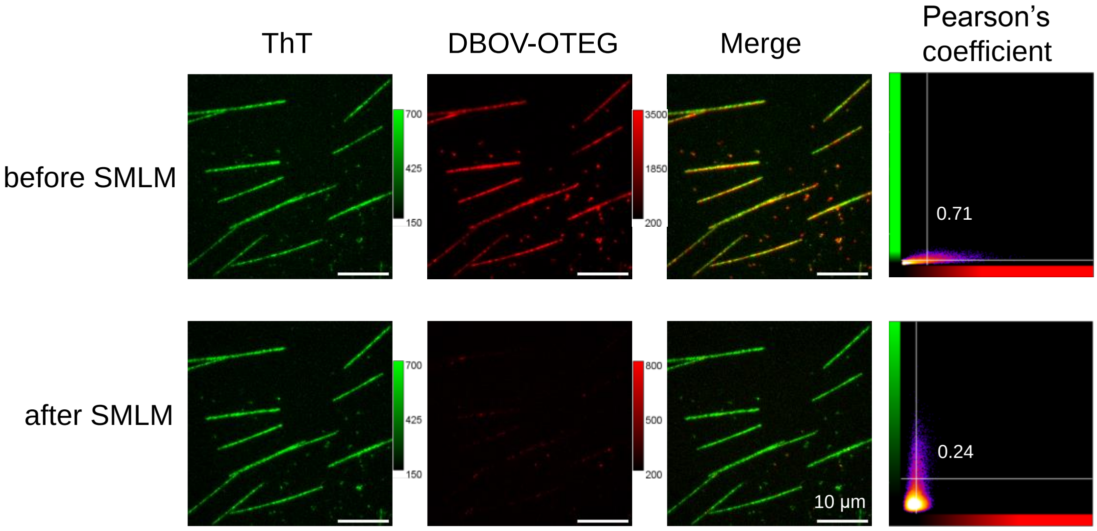

Figure S20. Wide-field images of amyloid fibrils labeled with ThT and DBOV-OTEG in air. The images on the top were taken before performing the SMLM. The images below were taken after performing SMLM. During SMLM, most of DBOV molecules were in dark state and only few of them were detected on each wide-field imaging frame. The parameters (exposure time and laser intensity) of microscope setting are the same when taking images before and after SMLM. The ThT was excited with 488 nm laser and the DBOV-OTEG was excited with 642 nm laser. The brightness scale bar of each image is showing on the right of the corresponding images. The length scale bar for all images is 10 μm.

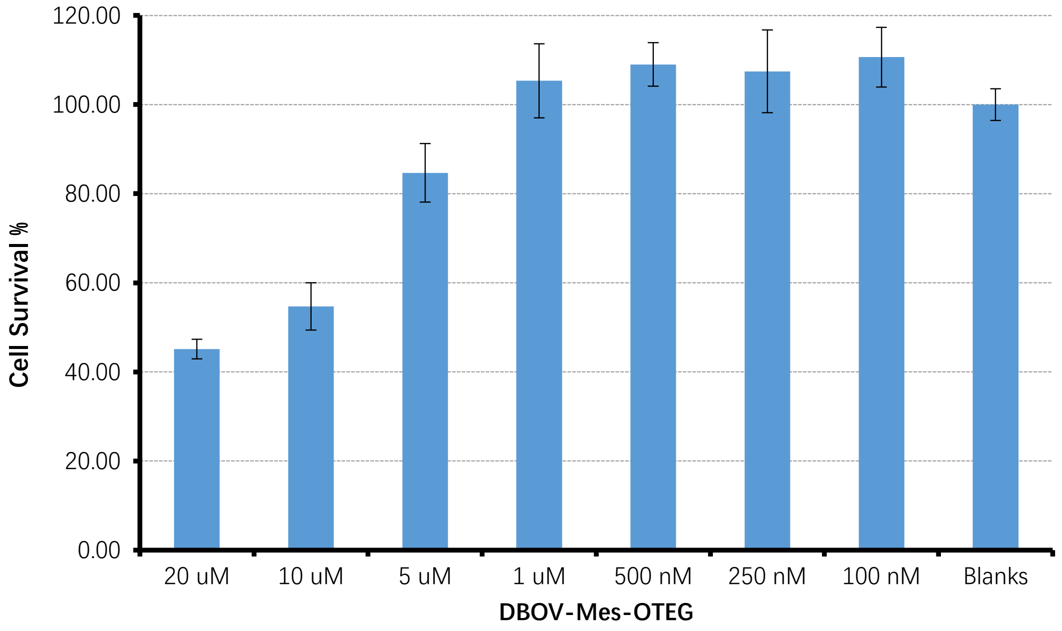

Figure S21. Viabilities of Hela cells after incubation with different concentrations of DBOV-OTEG for 24 h

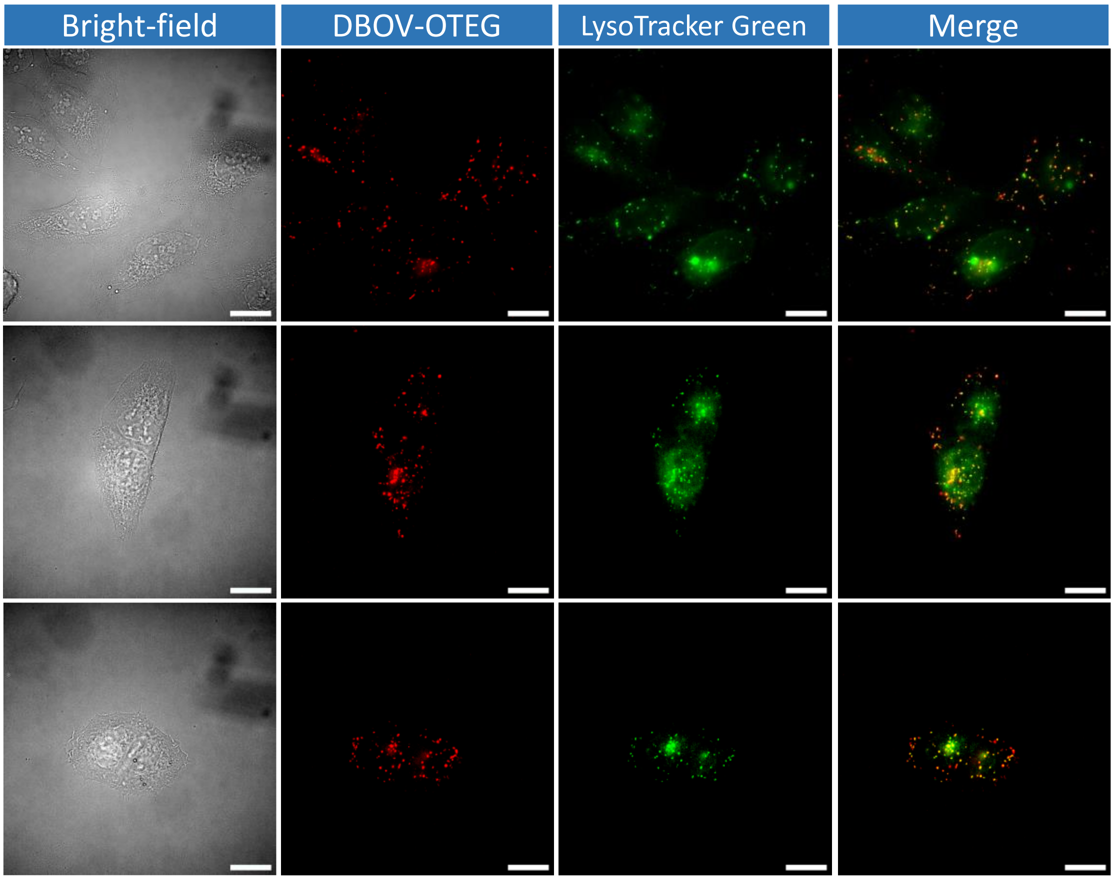

Figure S22. Conventional wide-field fluorescence images of live U2OS cells with DBOV-OTEG and LysoTracker Green. The U2OS cells were incubated with DBOV-OTEG (1 μM) for four hours in DMEM (supplement 10% FBS) and rinsed three times with DMEM, and LysoTracker Green (75 nM) in DMEM was added for 30 mins. The U2OS cells were washed twice with DMEM. Afterwards, DMEM (supplement 10% FBS) was added for imaging. Left: bright-field images. Middle (red channel): conventional wide-field fluorescence images (DBOV-OTEG). Middle (green channel): conventional wide-field fluorescence images (LysoTracker Green). Right: Merge images (DBOV-OTEG and LysoTracker Green). These images show that the DBOV-OTEG mainly accumulated into the lysosomes. The length scale bar for all images is 20 μm.

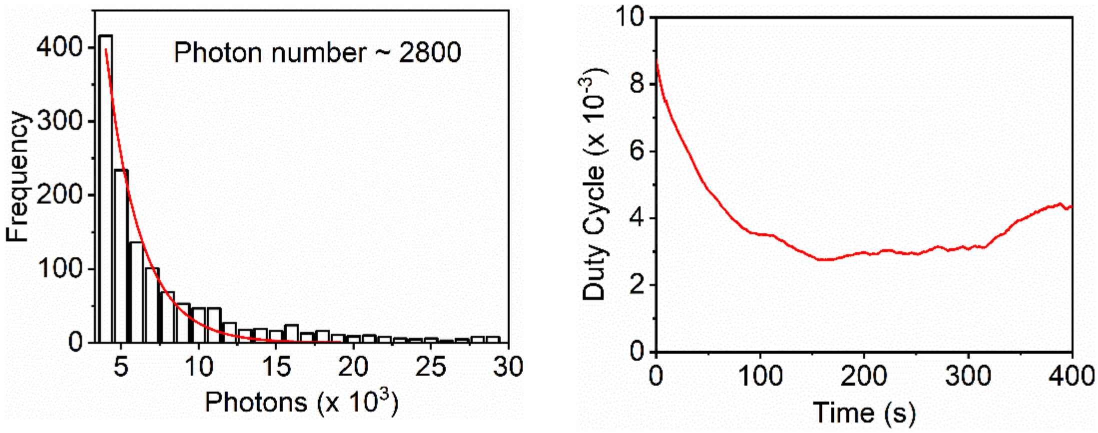

Figure S23. Blinking properties of single-molecule DBOV-azide in air, Left: Histogram of detected photons per switching event and single-exponential fit. Right: On-off duty cycle (fraction of time a molecule resides in its fluorescent state).

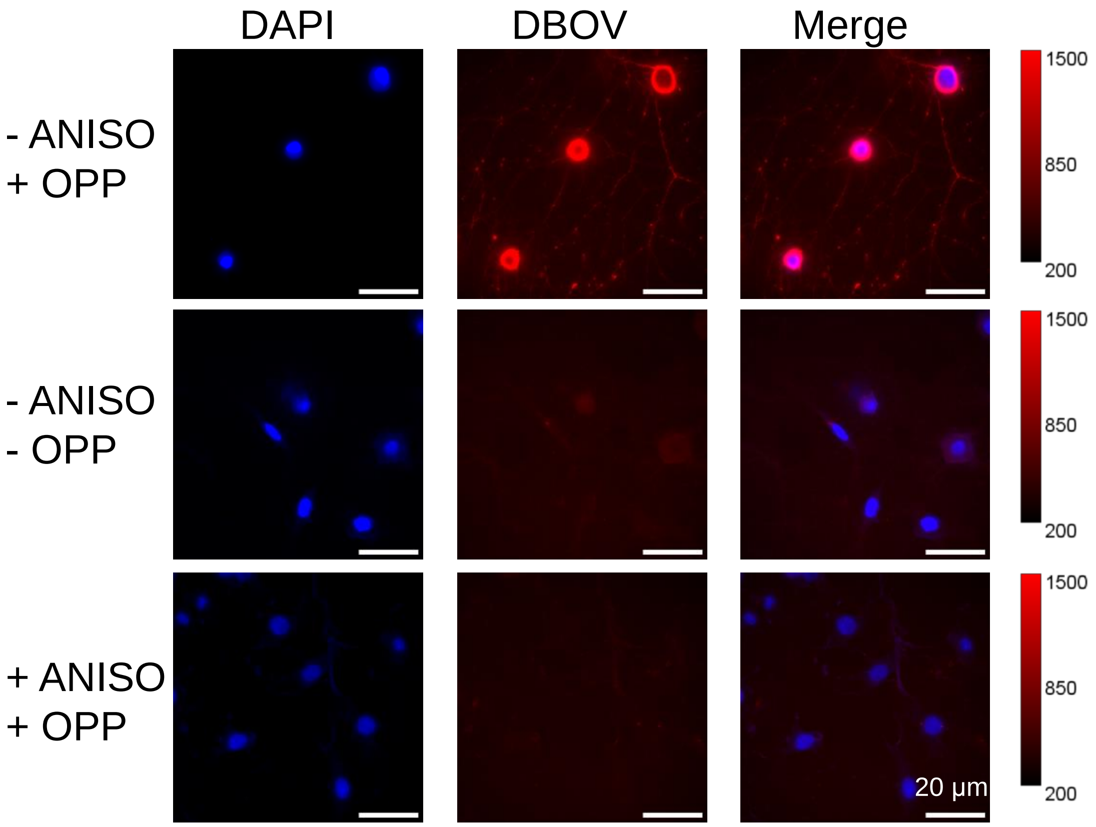

Figure S24. Wide-field images of neurons treated with anisomycin (ANISO), o-propargyl-puromycin (OPP) and DBOV-azide (DBOV) in PBS. The top panel displays neurons labeled OPP in absence of ANISO. Newly synthesized proteins tagged with OPP were clicked with DBOV-azide (in red). The panel in the middle displays untreated neurons, which were still subjected to click reaction with DBOV-azide. Finally, the bottom panel displays neurons treated with ANISO for 30 min first, then treated with OPP and clicked with DBOV-azide. The parameters (exposure time and laser intensity) of microscope setting are the same for the three samples. The DAPI was excited with 405 nm laser and the DBOV-azide was excited with 635 nm laser. The brightness scale bar of images of DBOV-azide channel is showing on the right of images. The length scale bar for all images is 20 μm.

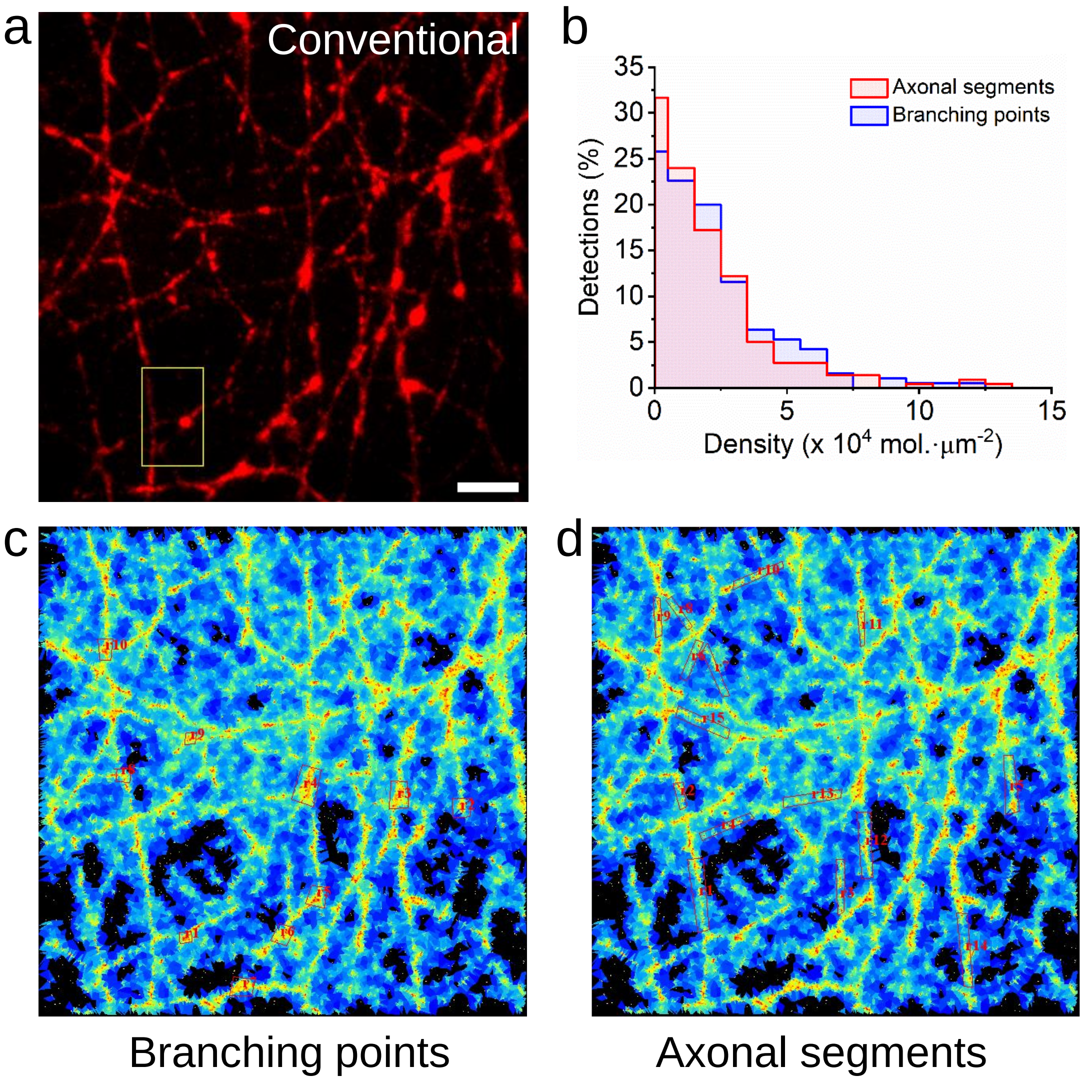

Figure S25. **a,** Conventional wide-field fluorescence image of neuronal networks treated with o-propargyl-puromycin (OPP) and DBOV-azide (DBOV) in PBS. The region of interest (ROI) in yellow is showed in Figure 4g in the main text. Scale bar: 5 μm. **b,** Distribution of the first-rank density (single-molecule localizations/μm^2) of punctas of linear axons (ROI in **d**) and branch points (ROI in **c**). **c,** Corresponding Voronoi diagram of **a**, the branching points are labeled for further analysis shown in Figure 4h, i and Figure S 25 b. **d,** Corresponding Voronoi diagram of **a**, the axonal segments are labeled for further analysis shown in Figure 4h, i and Figure S 25 b.

References

(1) Chen, Q.; Wang, D.; Baumgarten, M.; Schollmeyer, D.; Müllen, K.; Narita, A. Regioselective Bromination and Functionalization of Dibenzo[hi,st]Ovalene as Highly Luminescent Nanographene with Zigzag Edges. *Chem. - An Asian J.* **2019**, *14* (10), 1703–1707.
(2) Li, W. S.; Yamamoto, Y.; Fukushima, T.; Saeki, A.; Seki, S.; Tagawa, S.; Masunaga, H.; Sasaki, S.; Takata, M.; Aida, T. Amphiphilic Molecular Design as a Rational Strategy for

Tailoring Bicontinuous Electron Donor and Acceptor Arrays: Photoconductive Liquid Crystalline Oligothiophene-C60 Dyads. *J. Am. Chem. Soc.* **2008**, *130* (28), 8886–8887.
(3) Zhang, W.; Guo, C.; Liu, L.; Qin, J.; Yang, C. Naked-Eye Visible and Fluorometric Dual-Signaling Chemodosimeter for Hypochlorous Acid Based on Water-Soluble p-Methoxyphenol Derivative. *Org. Biomol. Chem.* **2011**, *9* (15), 5560–5563.
(4) Liu, X.; Chen, S.; Chen, Q.; Yao, X.; Gelléri, M.; Ritz, S.; Kumar, S.; Cremer, C.; Landfester, K.; Müllen, K.; Parekh, S. H.; Narita, A.; Bonn, M. Nanographenes: Ultrastable, Switchable, and Bright Probes for Super-Resolution Microscopy. *Angew. Chem. Int. Ed.* **2020**, *59* (1), 496–502.
(5) Tanaka, T.; Yamagami, T.; Nogami, T.; Minami, H.; Okubo, M. Preparation of Hemispherical Polystyrene Particles Utilizing the Solvent Evaporation Method in Aqueous Dispersed Systems. *Polym. J.* **2012**, *44* (11), 1112–1116.
(6) Ovesný, M.; Křížek, P.; Borkovec, J.; Švindrych, Z.; Hagen, G. M. ThunderSTORM: A Comprehensive ImageJ Plug-in for PALM and STORM Data Analysis and Super-Resolution Imaging. *Bioinformatics* **2014**, *30* (16), 2389–2390.
(7) Rieger B.; Stallinga S. The Lateral and Axial Localization Uncertainty in Super-Resolution Light Microscopy. *ChemPhysChem*, **2014**, *15* (4), 664–670.
(8) Levet, F.; Hosy, E.; Kechkar, A.; Butler, C.; Beghin, A.; Choquet, D.; Sibarita, J. SR-Tesseler : A Method to Segment and Quantify Localization-Based Super-Resolution Microscopy Data. *Nat. Methods* **2015**, *12* (11), 1065–1071.
